# Single-cell analysis of matrisome-related genes in breast invasive carcinoma: new avenues for molecular subtyping and risk estimation

**DOI:** 10.3389/fimmu.2024.1466762

**Published:** 2024-10-18

**Authors:** Lingzi Su, Zhe Wang, Mengcheng Cai, Qin Wang, Man Wang, Wenxiao Yang, Yabin Gong, Fanfu Fang, Ling Xu

**Affiliations:** ^1^ Department of Oncology, Yueyang Hospital of Integrated Traditional Chinese and Western Medicine, Shanghai University of Traditional Chinese Medicine, Shanghai, China; ^2^ The First Affiliated Hospital of Naval Military Medical University, Shanghai, China

**Keywords:** breast cancer, cellular matrix gene, single-cell sequencing, matrix score, molecular subtyping

## Abstract

**Background:**

The incidence of breast cancer remains high and severely affects human health. However, given the heterogeneity of tumor cells, identifying additional characteristics of breast cancer cells is essential for accurate treatment.

**Purpose:**

This study aimed to analyze the relevant characteristics of matrix genes in breast cancer through the multigroup data of a breast cancer multi-database.

**Methods:**

The related characteristics of matrix genes in breast cancer were analyzed using multigroup data from the breast cancer multi database in the Cancer Genome Atlas, and the differential genes of breast cancer matrix genes were identified using the elastic net penalty logic regression method. The risk characteristics of matrix genes in breast cancer were determined, and matrix gene expression in different breast cancer cells was evaluated using real-time fluorescent quantitative polymerase chain reaction (PCR). A consensus clustering algorithm was used to identify the biological characteristics of the population based on the matrix molecular subtypes in breast cancer, followed by gene mutation, immune correlation, pathway, and ligand-receptor analyses.

**Results:**

This study reveals the genetic characteristics of cell matrix related to breast cancer. It is found that 18.1% of stromal genes are related to the prognosis of breast cancer, and these genes are mostly concentrated in the biological processes related to metabolism and cytokines in protein. Five different matrix-related molecular subtypes were identified by using the algorithm, and it was found that the five molecular subtypes were obviously different in prognosis, immune infiltration, gene mutation and drug-making gene analysis.

**Conclusions:**

This study involved analyzing the characteristics of cell-matrix genes in breast cancer, guiding the precise prevention and treatment of the disease.

## Introduction

Breast cancer is currently the most common tumor in the world (1). The incidence of breast cancer is 11.7%, and the mortality rate is 6.9% worldwide, placing a heavy burden on human health and the health system. In addition to conventional surgical procedures, chemotherapy, radiotherapy, endocrine therapy (2–8), targeted therapy (9–12), and immunotherapy (13), breast cancer treatment strategies are increasingly considered by researchers and clinicians. Owing to the heterogeneity of tumor cells, improving the clinical efficacy of these treatment methods is complicated. Therefore, exploring approaches to improve the clinical effectiveness of breast cancer treatments is essential. Single-cell sequencing technology focuses on individual cells, performing uniform amplification of genetic material from single cells, followed by library preparation and sequencing. Finally, data analysis is conducted on the genome or transcriptome of individual cells. The technical principles mainly include three aspects: single-cell isolation, amplification sequencing, and data analysis. This technology has advantages in revealing cell characteristics, identifying tumor heterogeneity, and understanding the microenvironment (14), and provide researchers with more decision-making information.

Cells play a crucial role in life processes. Studies (15) have shown that during cell migration, intense nuclear deformation causes nuclear membrane rupture, accompanied by DNA damage, and researchers (16) have found that DNA damage and nuclear membrane rupture concurrently promote the cellular production of invasive phenotypes, which might promote the progression of breast tumors. An increasing number of researchers have recently focused on extracellular structures. The extracellular matrix (ECM) is a complex dynamic grid structure comprising macromolecules secreted by cells into the extracellular stroma, which is composed of an interstitial matrix and a basement membrane, constituting more than one-third of the body mass (17). It is an essential component of the biological cell microenvironment, cell proliferation, and survival. As a significant participant in differentiation and migration, the ECM has long been ignored as an inert framework; however, an increasing number of studies have found that the cytoplasmic matrix is closely related to many diseases, particularly tumors (18, 19). Despite significant progress in deciphering breast cancer at the whole-genome level, the mechanisms of matrix body genes in breast cancer have not yet been studied. Stromal-specific tumor biology involves integrating several RNA-sequencing (RNA-seq) and single-cell RNA seq (scRNA-seq) data, cell type deconvolution, ligand-receptor interaction analysis, and rich biological pathways to obtain the biological characteristics of matrix genes. A model was established to identify malignant breast cancers based on matrix gene expression. Understanding the characteristics of matrix genes could offer valuable insights into the diagnosis of poor prognosis and the development of treatment strategies for breast cancer.

With the deepening of the human understanding of tumors, researchers have realized that all cell types in the tumor microenvironment markedly influence tumors, among them, CD8 T cells are the most valued by researchers, with the main function of killing tumor and other pathological cells (20). CD4 T cells, due to their numerous subtypes, have diverse roles; on one hand, they can help tumors escape and suppress anti-tumor immune responses, while on the other hand, they can promote anti-tumor immune responses and inhibit tumor growth (21). An increasing number of researchers have found that other cells, such as dendritic cells and natural killer cells, play a key role in the initiation, regulation, and maintenance of anti-tumor immune responses (22, 23). Therefore, paying attention to the infiltration of immune cells is of great significance for tumor research. Some studies (24) have found that immune infiltration in patients with breast tumors is associated with clinical prognosis. Improving breast cancer treatment requires a comprehensive understanding of the biological features of the breast tumor microenvironment and its influencing factors. Studies on the relationship between cell-matrix genes and immune infiltration are unavailable; therefore, we analyzed immune infiltration in the molecular subtypes of matrix genes to observe the immune infiltration characteristics across different molecular subtypes and offer insights for clinical treatment.

To investigate the correlation between breast cancer and cell-matrix genes, we first used biological data on breast tumors and clinical survival data from various databases, such as the Cancer Genome Atlas (TCGA). Elastic net penalty logistic regression was used to pinpoint highly correlated differentially expressed matrix genes and construct a stromal risk regression model for subsequent analyses, which was used based on the Monte Carlo consensus clustering algorithm to identify different matrix-related molecular subtypes, and subsequently through gene ontology (GO)/Kyoto Encyclopedia of Genes and Genomes (KEGG), immune infiltration, receptor-ligand, and other analytical methods to show the biological characteristics of different molecular subtypes. Based on the above, the study guides the clinical study of breast cancer and helps more patients with breast cancer to benefit from survival.

## Materials and methods

### Technical overview

This study investigates the prognostic value of matrix genes in breast invasive carcinoma (TCGA-BRCA) using data from the TCGA database. We downloaded the dataset consisting of 1,222 samples, integrated clinical data, and filtered for 1,109 patients with complete survival and TNM staging information.

Differential expression analysis was conducted using the limma package to identify differentially expressed genes (DEGs) linked to patient survival, categorizing patients into long-term (≥1 year) and short-term (<1 year) survival groups. Gene Ontology (GO) and KEGG pathway enrichment analyses were performed on these DEGs to understand their biological roles.

We developed a risk signature utilizing elastic net penalized logistic regression, optimizing the model to predict patient outcomes based on gene expression profiles. Each patient received a matrix risk score, enabling classification into high- and low-risk groups, followed by survival analysis using Kaplan–Meier curves.

**Figure 1:**
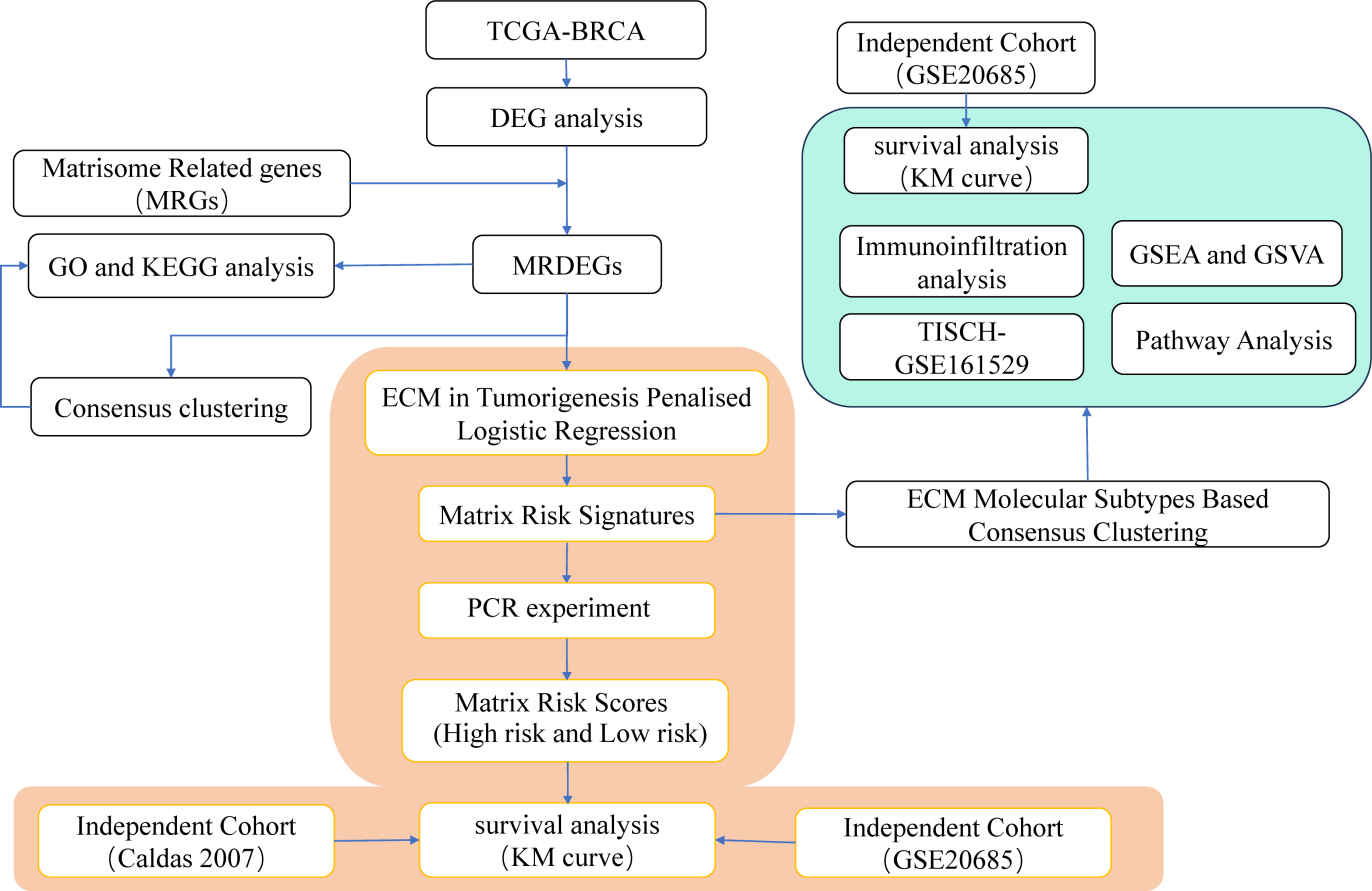
Work Flow. GO, Gene Ontology; KEGG, Kyoto Encyclopedia of Genes and Genomes; DEGs, differentially expressed genes; MRDEGs, matrisomal-related differentially expressed genes; TISCH: Tumor Immune Single-cell Hub; BRCA, Breast invasive carcinoma; TCGA, The Cancer Genome Atlas; GSEA, Gene Set Enrichment Analysis; GSVA, Gene Set Variation Analysis; ECM, extracellular matrix.

To identify molecular subtypes, we implemented consensus clustering on MRDEG expression and validated results with independent datasets. We also assessed immune cell infiltration using the CIBERSORT algorithm. Pathway activity was analyzed with GSVA, focusing on hallmark pathways. Additionally, ligand-receptor interactions were examined to explore signaling dynamics in the tumor microenvironment. Statistical analyses were performed in R, with p<0.05 considered significant **Figure 1**.

### Data download

We downloaded the breast invasive carcinoma (BRCA) dataset, TCGA-BRCA (n=1,222), from the TCGA database (25) using the TCGAbiolinks package (26). The data type was selected as count and converted to FPKM format. In addition, we obtained clinical data corresponding to the matched samples of the TCGA BRCA dataset from the TCGA GDC^1^ official website, including age, survival status, follow-up time, and tumor stage. Our study excluded patients with no survival information and incomplete TNM staging information, and 1,109 patients were included in our subsequent analyses.

To examine the gene mutation status of TCGA-BRCA patients, we acquired ‘Masked Somatic Mutation’ data from the official website of TCGA. The obtained data served as the somatic mutation dataset for patients with breast cancer. We preprocessed the data using VarScan software and visualized the somatic mutations of patients using the maftools package (27). We obtained the body genes from the study of Naba et al. (28), which contains 1,062 matrix body genes, and the specific information is presented in **Supplementary Table S1** and **Table 1**.

In addition, we assessed the Molecular Signature Database (29) (MSigDB) ^2^. The 50 Hallmark gene sets were obtained from “h.all.v2023.1.Hs.symbols.gmt” on the database website, from “c2.cp.kegg.v 7.4.symbols.gmt” file to obtain the KEGG pathway gene set for subsequent Gene Set Enrichment Analysis (GSEA).

To further validate our approach, we acquired a set of scRNA-seq data, GSE161529, from the Tumor Immune Single-cell Hub 2^3^ database (30). Additionally, we obtained an independent validation set for breast cancer, UCSC (Caldas 2007), from the Xena platform ^4^, and another independent validation set, GSE20685, from the Gene Expression Omnibus (GEO) database.

### Differentially expressed genes related to breast cancer survival

To elucidate the potential mechanism of gene action and related biological characteristics influencing the prognoses of patients with breast cancer, we first divided patients with an overall survival of >1 year into the long survival group according to their prognoses. Patients aged<1 year were classified into the short-term survival group. Genes in different groups were subjected to differential analysis using the limma package. Genes with an absolute value of log fold change (|logFC|) >0.25 and a p-value<0.05 were considered as differentially expressed genes (DEGs) associated with prognosis in patients with breast cancer. To obtain breast cancer-related matrix body-related DEGs (microsomal-related (MR) DEGs), we compared the DEGs obtained from the differential analysis of TCGA-BRCA datasets with matrix body-related genes (microsomal-related genes, MRGs) at the intersection and drew a Venn diagram. The findings from the differential analysis were visualized using the R package ggplot2 to generate a volcano plot and the R package pheatmap to create a heatmap.

### GO and pathway (KEGG) enrichment analysis

GO analysis (31) is a standard method for large-scale functional enrichment studies, including biological processes, cell components, and molecular functions. KEGG (32) is a database containing information on genomes, biological pathways, diseases, and drugs. We used R-package clusterProfiler (33) to analyze the GO and pathway (KEGG) enrichment of differentially expressed matrix genes, and the screening criteria for entries were adj. p<0.05. An FDR value (q-value)<0.25 was considered statistically significant, and the correction method of p-value was Benjamini–Hochberg.

### Construction of stromal body risk characteristics in breast cancer

We performed risk signature selection on matrix genes differentially expressed between long- and short-term survival in the TCGA-BRCA dataset. We used Elastic Net penalized logistic regression to select highly correlated differentially expressed matrix genes based on the correlation between long and short survival. The elastic net penalized logistic regression was implemented using the glmfit function in the R package glmnet, where the parameter alpha was set to 0.5. Using α=0.5 in penalized logistic regression is to combine the advantages of Lasso (L1 penalty) and Ridge (L2 penalty) regression, allowing for both variable selection and handling of feature correlation issues. We select the shrinkage coefficient λ through cross-validation, specifically by finding a λ value that minimizes prediction error and ensuring that this value is within one standard error range, which helps prevent overfitting and improves the model’s predictive ability on new data. We initially normalized the expression profiles of the samples in the TCGA-BRCA dataset using the Z-scale. Subsequently, we used the createDataPartition function in the caret package to split the samples into training and test sets with 80% and 20% allocations, respectively. In this study, we developed an elastic net penalized logistic regression model using only the training set. The shrinkage coefficient (lambda) was selected as a value within a standard error range to minimize the cross-validation prediction error rate, and the model feature with a minor prediction error was selected as the final marker gene. To generate a gene-based matrix risk score for the samples, Firth’s correction was used to calculate the odds ratios using the logistic function in the logistic package. The matrix of each sample risk score is the sum of the product of the risk ratio and the expression value of each marker gene; that is $\text{Matrix Risk Score }=\sum_{\text{i}=1}^{\text{n}}{\text{z}}_{\text{i}}{\text{β}}_{\text{i}}$ , where n is the length of the marker gene, ${\text{z}}_{\text{i}}$ is the expression of gene i, and ${\text{β}}_{\text{i}}$ is the log-odds ratios of gene i. Subsequently, according to the dataset, the patient matrix risk score was used to determine the best grouping through the surv_cutpoint function and divide patients into high- and low-risk groups. The Kaplan–Meier test was used to compare differences in overall survival among the different sample groups.

### Identification of stromal molecular subtypes in breast cancer

We used a consensus clustering algorithm based on Monte Carlo references (Monte Carlo reference-based consensus clustering, M3C) (34) based on MRDEG expression to identify matrix-associated molecular subtypes. M3C is a consensus-clustering algorithm that involves using Monte Carlo simulations to mitigate the overestimation of K and effectively reject the null hypothesis of K=1. Real data were compared to eliminate bias, and statistical tests for the presence of structures were used to correct for inherent bias in consensus clustering. The optimal number of clusters K has the largest relative cluster stability index, the proportion of Monte Carlo P value is<0.05, and the fuzzy clustering score (Proportional Ambiguous Clustering, PAC) is the smallest. To confirm the accuracy of consensus clustering, the results were validated using a validation set. Subsequently, the R package ggpubr was used to generate a box plot, with the sample cluster labels as groups. Group differences were assessed for statistical significance using the Wilcoxon rank-sum test, with a p-value<0.05 indicating statistical significance.

Gene mutation analysis of stromal molecular subtype populations in different breast cancers

Breast cancer data were downloaded from GDC, and all non-synonymous mutations were selected for downstream analysis. R package map tools were used to display the related gene mutations, the biological functions affected by the mutations, and the classification of potentially druggable genes in different groups of breast cancer stromal molecular phenotype characteristics.

### Immune-related analysis of the population of stromal molecular subtypes in different breast cancers

To identify the underlying molecular mechanisms of different stromal molecular subtypes in patients with breast cancer, we first performed ESTIMATE (35) on the TCGA-BRCA dataset. We analyzed and calculated four tumor-related scores, namely the matrix score, immune score, tumor purity, and ESTIMATE score, the immune score and matrix score calculated based on the ESTIMATE algorithm can facilitate the quantification of immune and matrix components in tumors; in this algorithm, immune and matrix scores are calculated by analyzing the specific gene expression characteristics of immune and matrix cells to predict the infiltration of non-tumor cells. Subsequently, the CIBERSORT algorithm (36) was applied to assess the infiltration status of immune cells within integrated datasets of various tumor samples. Next, differences in immune cell infiltration among different tumor subgroups were examined using the Wilcoxon test. Statistical significance was set at p<0.05. CIBERSORT^5^ involves using linear support vector regression and serves as an R/web tool for deconvoluting expression matrices of human immune cell subtypes. It is used to evaluate the infiltration status of immune cells in sequenced samples using a gene expression signature set of 22 known immune cell subtypes. In addition, we analyzed the differential expression of immune checkpoint genes across different matrix molecular subtypes.

### Path analysis

Seen in different subtypes of matrix molecules, we performed pathway enrichment analysis based on the 50 hallmark and C2 oncogenic pathways in patients with different subtypes. Pathway activity was assessed for each sample using the GSVA algorithm, and differentially active pathways were identified using a t-test.

### Ligand-receptor interaction analysis

We annotated the genes in the RNA-seq dataset as ligands and receptors using a curated database of human ligand-receptor pairs previously published by Ramilowski et al. (37). We retained only ligands corresponding to core matrix genes identified by Naba et al. (28) for subsequent analyses. The interaction score between a core matrix gene and its receptor was computed as the product of the expression values of the ligand (core matrix gene) and its cognate receptor in each sample. We identified the relative enrichment of ligand-receptor interaction scores among samples of different matrix subtypes using the Wilcoxon test and visualized the results using Circos.

### Single-cell analysis

All single-cell data analyses and integrations were performed using R software Seurat v 4.0.6. Two-cell quality control was implemented using the R Scrublet package. Cells with fewer than 300 genes, as revealed by single-cell sequencing, were deleted through quality control. Similarly, cells with more than 20% of the mitochondrial gene reads were deleted. The normalization and standardization of each sample data were realized through principal component analysis, and the inter-batch difference between samples was determined using the Harmony package. We used the t-distributed stochastic neighbor embedding algorithm to reduce dimensionality and visualize the single-cell data. The ECM scores of different cells were calculated using the AddModuleScore function.

### qPCR

For qPCR, total RNA was extracted using RNAiso Plus (TaKaRa, Japan), followed by reverse transcription using PrimeScript™ RT Master Mix (TaKaRa, Japan). qPCR was conducted using AceQ Universal SYBR qPCR Master Mix (Vazyme, China). The primer sequences are listed in **Supplementary Table S2.**

### Statistical analysis

All data processing and statistical analyses were conducted using the R software^6^. To compare two groups of continuous variables, the independent Student’s t-test was used to assess the statistical significance of normally distributed variables, whereas the Mann–Whitney U test was used for non-normally distributed variables. The U-test (i.e., the Wilcoxon rank-sum test) was used to analyze the differences among non-normally distributed variables. The chi-square or Fisher’s exact test was used to compare and analyze the statistical significance of categorical variables between the two groups. The survival package in R was used for survival analysis, using Kaplan–Meier survival curves to illustrate the survival differences. The significance of the survival time difference between the two patient groups was evaluated using a log-rank test. Univariate and multivariate Cox analyses were performed using the survival package in the R software. All statistical p-values were two-sided, and p<0.05 is considered statistically significant.

### R language

Detailed R packages can be found in **Supplementary Table S3**.

**Table 1:** Datasets accessed in this study.

Cohort	Data type	Source	Reference
TCGA-BRCA	RNAseq	TCGAbiolinks	Reference (25, 26)
Breast cancer	RNAseq	Gene Expression Omnibus GSE20685	Reference (38)
Breast cancer	RNAseq	UCSC xene	Reference (39)
Cell types from scRNAseq	scRNAseq	h5 files and Signature Matrix	Reference (40)
Data type			
Extracellular matrix gene set	Gene	Manuscript	Reference (28)
Cell types	scRNAseq	TISCH database	Reference (30)

## Results and discussion

### Data source

### Identification of differentially expressed matrix genes associated with breast cancer prognosis

**Figure 2:**
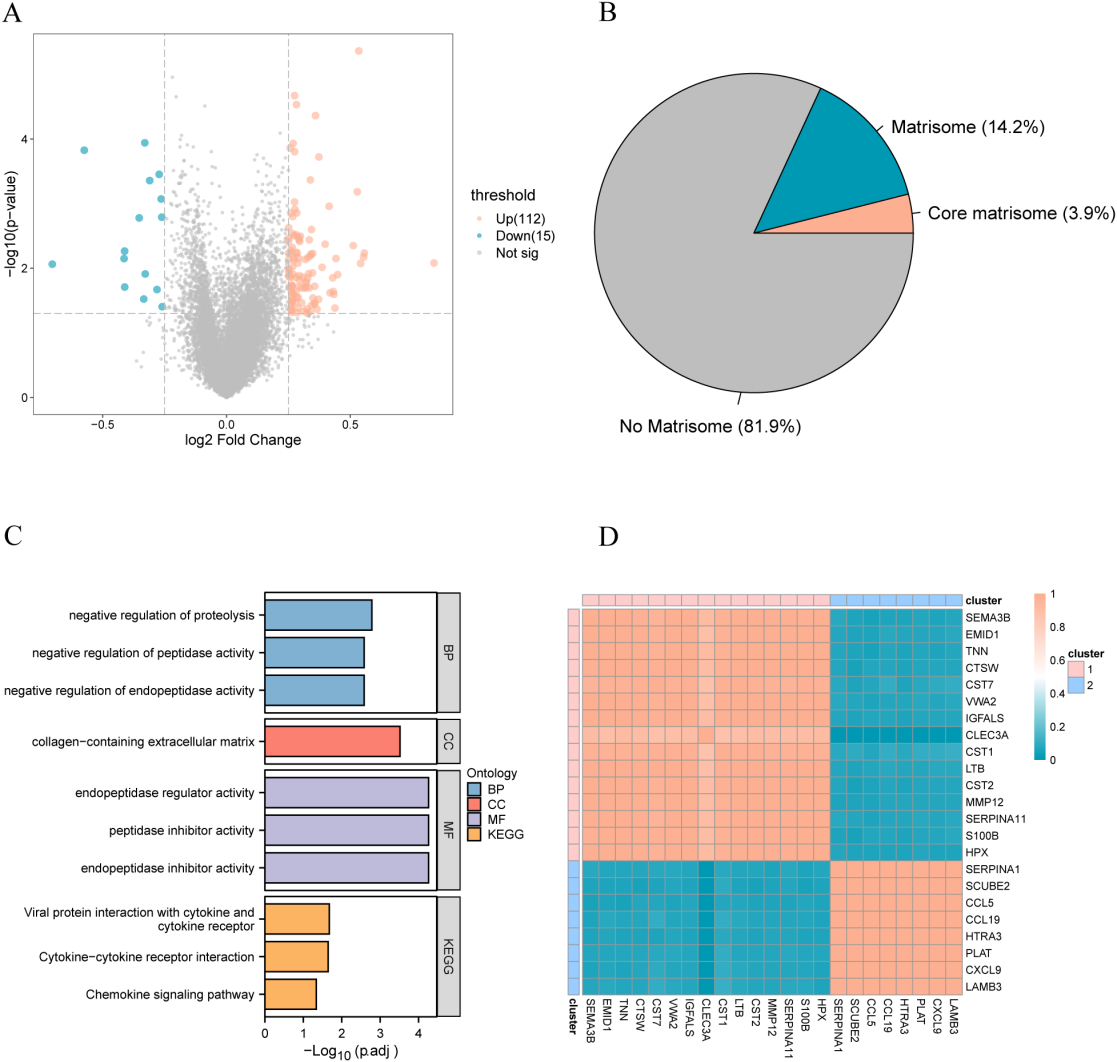
**(A)** The volcano plot of differential analysis, where red represents upregulated genes and blue represents downregulated genes; **(B)** The proportion of differential plastid genes, with red indicating the proportion of core plastid genes and blue indicating the proportion of other plastid genes; **(C)** Functional enrichment analysis of differentially expressed plastid genes; **(D)** Correlation heatmap of differentially expressed plastid genes. GO, Gene Ontology; BP, biological process; CC, cellular component; MF, molecular function; KEGG, Kyoto Encyclopedia of Genes and Genomes.

To further explore the underlying molecular mechanisms affecting the prognosis of patients with breast cancer, we conducted differential gene expression analysis on the complete TCGA-BRCA dataset to identify genes that were differentially expressed between patients in the long and short survival groups. Genes with an absolute value of log fold change (|logFC|) >0.25 and a p-value<0.05 were considered as DEGs associated with prognosis in patients with breast cancer, and 127 DEGs were identified (**Figure 2A**). Differential analysis revealed that 18.1% of the DEGs were stromal (**Figure 2B**). Of these genes, 3.9% were core matrix, and 14.2% were matrix-related. Subsequently, through Gene Ontology (GO) and KEGG analysis, biological processes and functions related to differentially expressed genes were identified. Among them, red represents biological processes, purple represents cellular components, blue represents molecular functions, and orange represents KEGG pathways. The p-values for all enriched functions are presented in the form of -log10(padj). In GO functional enrichment analysis, the analysis revealed the enrichment of biological processes associated with protein metabolism, including the negative regulation of endopeptidase activity, peptidase activity, and proteolysis. KEGG enrichment analysis indicated that the DEGs were associated with cytokines, including cytokine-cytokine receptor interaction and the chemokine signaling pathway (**Figure 2C**).

Recent advancements have underscored that individual matrix molecules rarely operate independently but as integral components within a dynamic three-dimensional supramolecular network comprising structurally and functionally integrated matrix constituents (41). We performed a correlation analysis of differentially expressed matrix genes in patients with breast cancer to determine whether these genes are also regulated in the disease (**Figure 2D**). Unsupervised clustering revealed two significant stromal body gene clusters related to somatic genes.

Volcano map display of differential analysis, in which red is the gene with up-regulated expression, and blue is the gene with down-regulated expression; (B) The proportion of differential matrix genes, red is the proportion of core matrix genes, and blue is the gene proportion of other matrix bodies; (C) Functional enrichment analysis of differentially expressed matrix genes; (D) Correlation heat map of differentially expressed matrix genes. GO, Gene Ontology; BP, biological process; CC, cellular component; MF, molecular function; KEGG, Kyoto Encyclopedia of Genes and Genomes.

**Figure 3:**
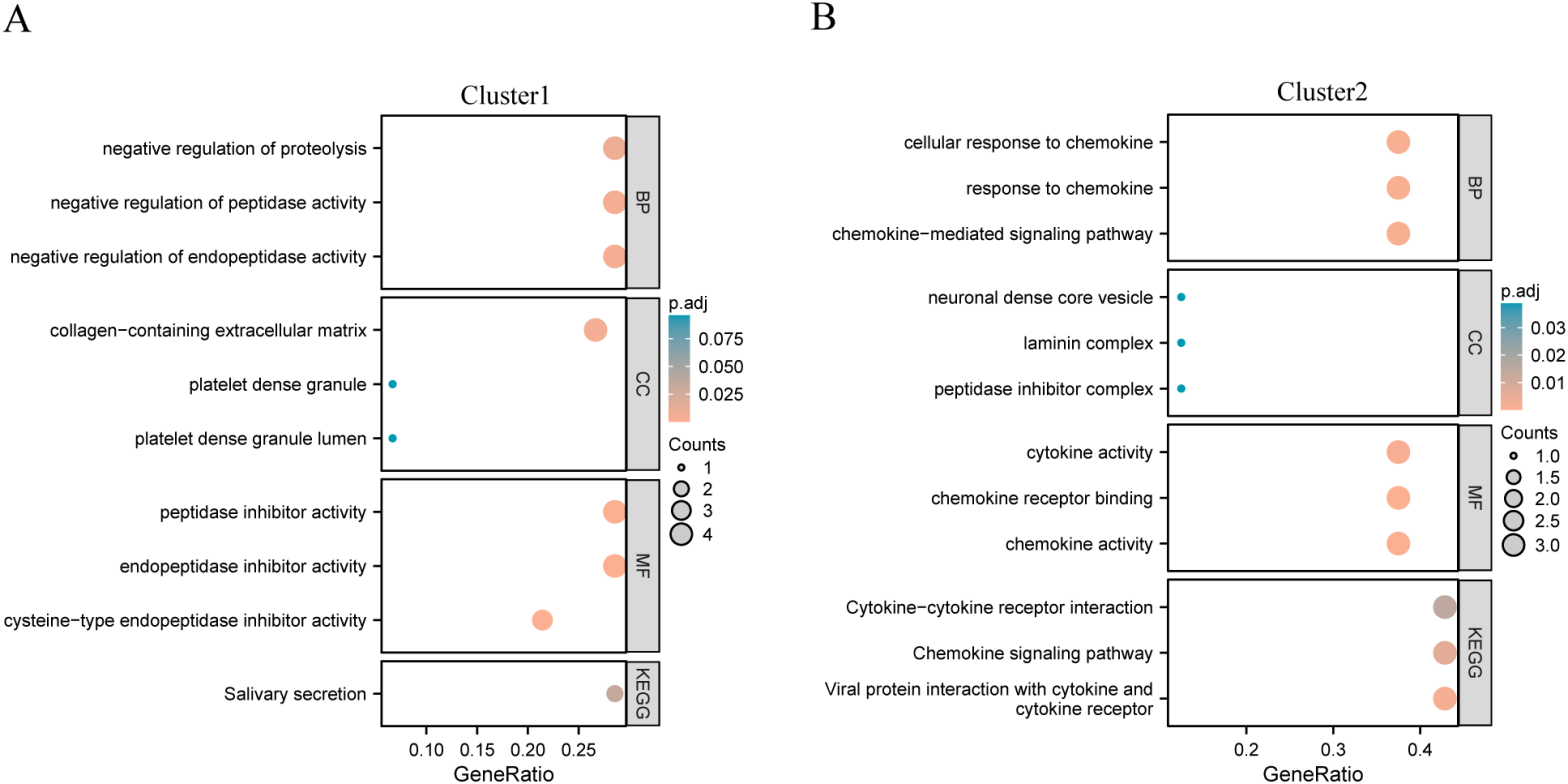
KEGG functional enrichment analysis of different gene clusters. **(A)** Gene cluster 1 enrichment results display; **(B)** Gene cluster 2 enrichment results display.

To explore the potential biological functions of different gene clusters, we performed GO and KEGG functional enrichment analyses on these two gene clusters. Gene Cluster 1 was primarily enriched in salivary secretion (**Figure 3A**), whereas gene Cluster 2 was primarily enriched in viral protein interactions with cytokines and cytokine receptors, chemokine signaling pathways, and cytokine-cytokine receptor interactions (**Figure 3B**). Concerning functional enrichment analysis with GO, gene Cluster 1 was found to be primarily enriched in the negative regulation of proteolysis and peptidase activity (**Figure 3A**). Gene Cluster 2 was primarily enriched in the chemokine-mediated signaling pathway, response to chemokines, and cellular response to chemokines (**Figure 3B**).

The enrichment result display of gene cluster1; (B) The enrichment result display of gene cluster2. GO, Gene Ontology; BP, biological process; CC, cellular component; MF, molecular function; KEGG, Kyoto Encyclopedia of Genes and Genomes.

### Construction of stromal body risk characteristics in breast cancer

**Figure 4:**
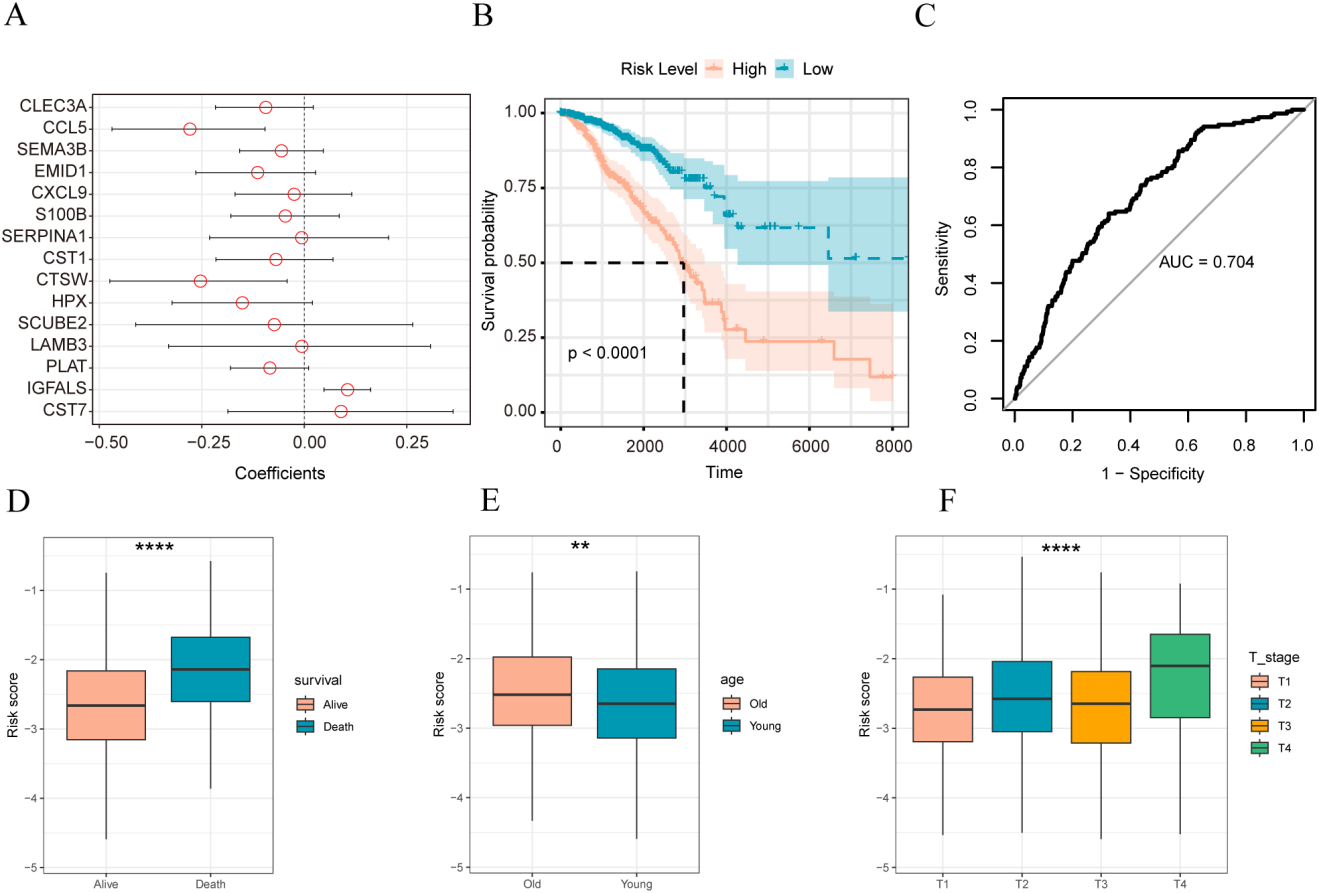
Construction of stromal body risk signature in breast cancer **(A)** Coefficient of genes in stromal risk signature; **(B)** K-M survival curve of high and low stromal risk score sample group; **(C)** ROC plot of stromal risk feature predicting patient prognosis; **(D)** stromal risk score in Distribution boxplots between living and dead patients; **(E)** distribution boxplots of stromal body risk scores between older and younger patients; **(F)** distributions of stromal body risk scores among patients with different T stages box plot. The symbol ** is equivalent to p < 0.01; the symbol **** is equivalent to p < 0.0001.

We identified 15 matrix-related marker genes using the elastic net penalized logistic regression method. To generate a matrix gene-based risk score for the samples, we used Firth’s correction to calculate the odds ratios(**Figure 4A**) and the logistic function in the logistic package. Subsequently, patients were divided into high-and low-risk groups based on their matrix risk score. The Kaplan–Meier test was used to compare the differences in overall survival among the different sample groups. Survival analysis showed that the constructed stromal body risk signature could be used to accurately distinguish and predict patient prognosis (**Figures 4B, C**). Furthermore, significant differences were found between patients with different clinical characteristics. For example, in patients with breast cancer who died, it was significantly higher (**Figure 4D**); in older patients, it was also higher than that in younger patients (**Figure 4E**) and significantly lower in patients with T1 stage disease (**Figure 4F**).

**Figure 5:**
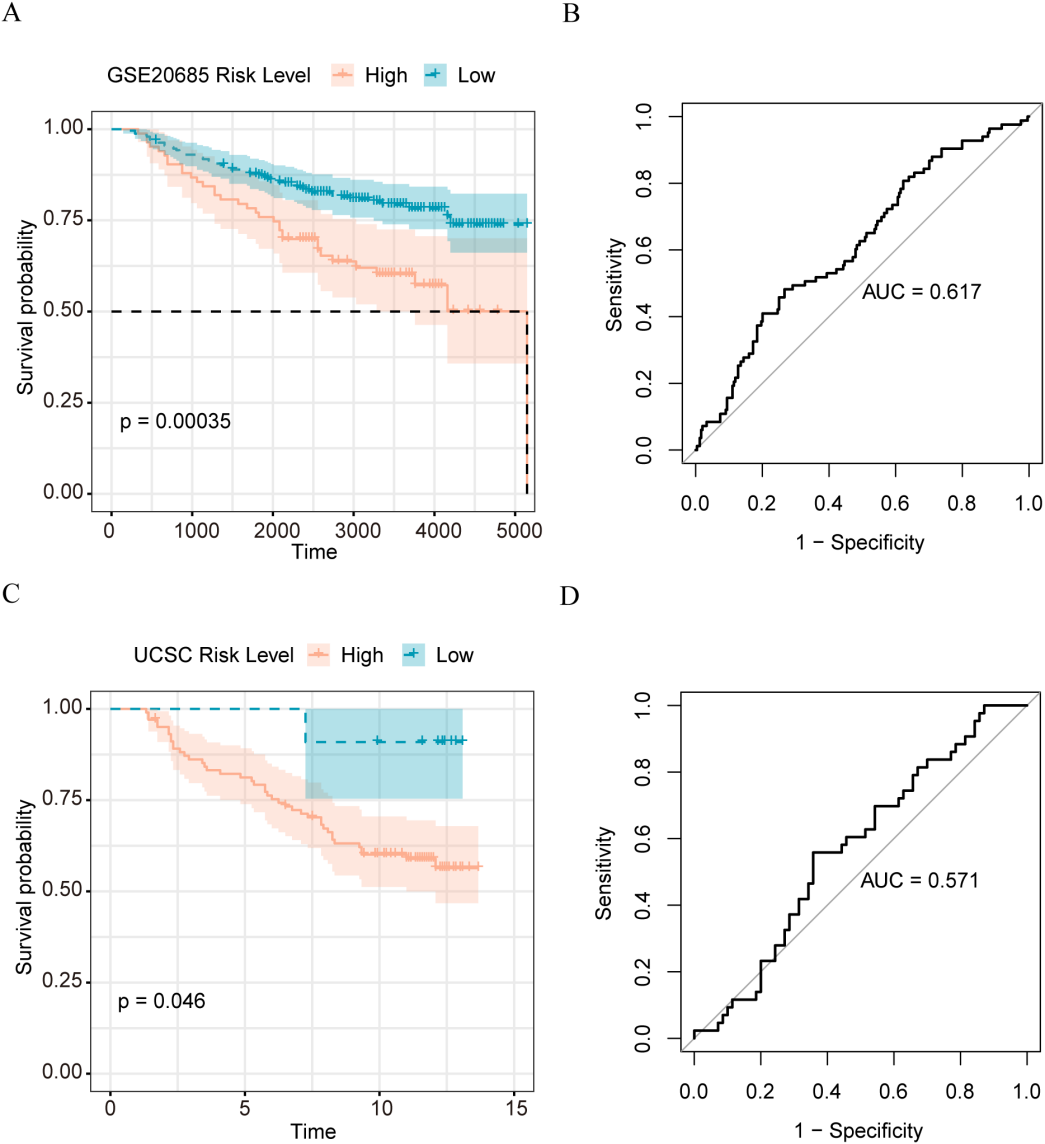
Validation of the performance of matrix body risk features **(A)** K-M survival curve of high and low stromal body risk score sample group in GSE20685 dataset; **(B)** ROC plot of stromal body risk characteristics in GSE20685 data set predicting patient prognosis; **(C)** UCSC K-M survival curve of high and low stromal body risk score sample group in Caldas 2007 dataset; **(D)** UCSC Caldas 2007 data set stromal body risk characteristics predict patient prognosis ROC plot.

To verify the effectiveness of our model, we applied our matrix body risk model to the GSE 20685 dataset, and the UCSC results of survival analysis on the Caldas 2007 dataset showed that our model could be used to significantly distinguish patients with breast cancer with different prognoses in the independent validation set (**Figures 5A, C**). The Receiver Operating Characteristic (ROC) curve analysis demonstrated that our model has a certain prognostic predictive ability and may have some clinical reference value; the Area Under Curve (AUC) of the GSE20685 dataset was 0.617 (**Figure 5B**), and that of the UCSC Caldas 2007 dataset was 0.571 (**Figure 5D**).

**Figure 6:**
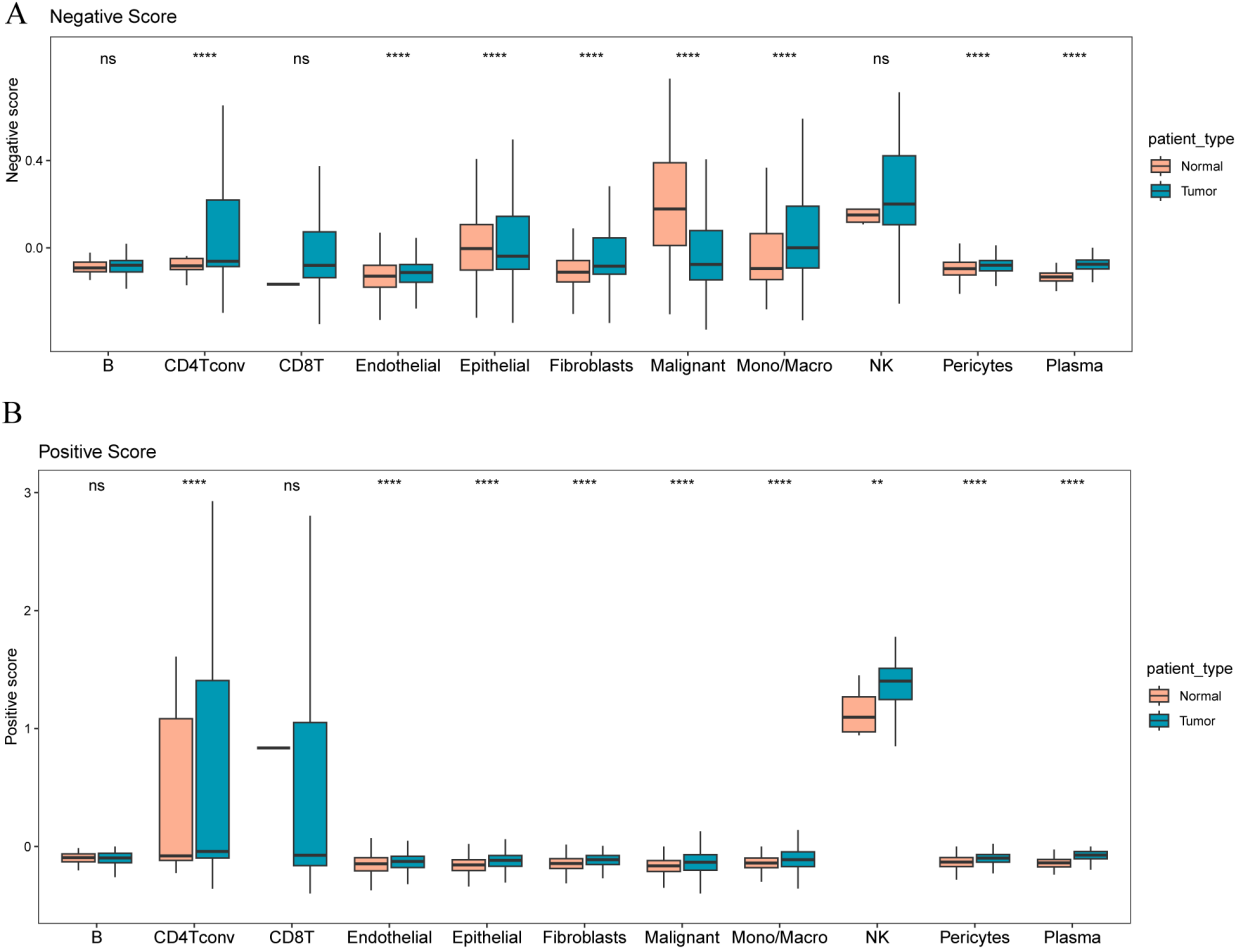
Distribution of different risk signature gene scores in tumor and paracancerous samples **(A)** Distribution of negative scores in different cell types in tumor and paracancerous samples; **(B)** Distribution of positive scores in different cell types in tumor and paracancerous samples. The symbol ns (not statistically significant) is equivalent to p≥0.05, no statistical significance; the symbol ** is equivalent to p < 0.01; the symbol **** is equivalent to p < 0.0001.

To explore which cell types express the marker genes we identified for constructing our stromal risk signature based on cell annotation information from a single-cell dataset (GSE 161529), we calculated positive ratios (positive score) and the enrichment degree of stromal risk genes with a negative ratio (negative score). CD4 Tconv, endothelial cells, epithelial cells, fibroblasts, malignant cells, Mono/Macro, pericytes, and plasma cells differed significantly between tumor and normal cells in the negative score (**Figure 6A**), and CD4 Tconv, endothelial cells, epithelial cells, fibroblasts, malignant cells, mono/macrophages, NK cells, pericytes, and plasma cells were significantly different between tumor and normal cells in the positive score (**Figure 6B**).

### Identification of stromal molecular subtypes in breast cancer

**Figure 7:**
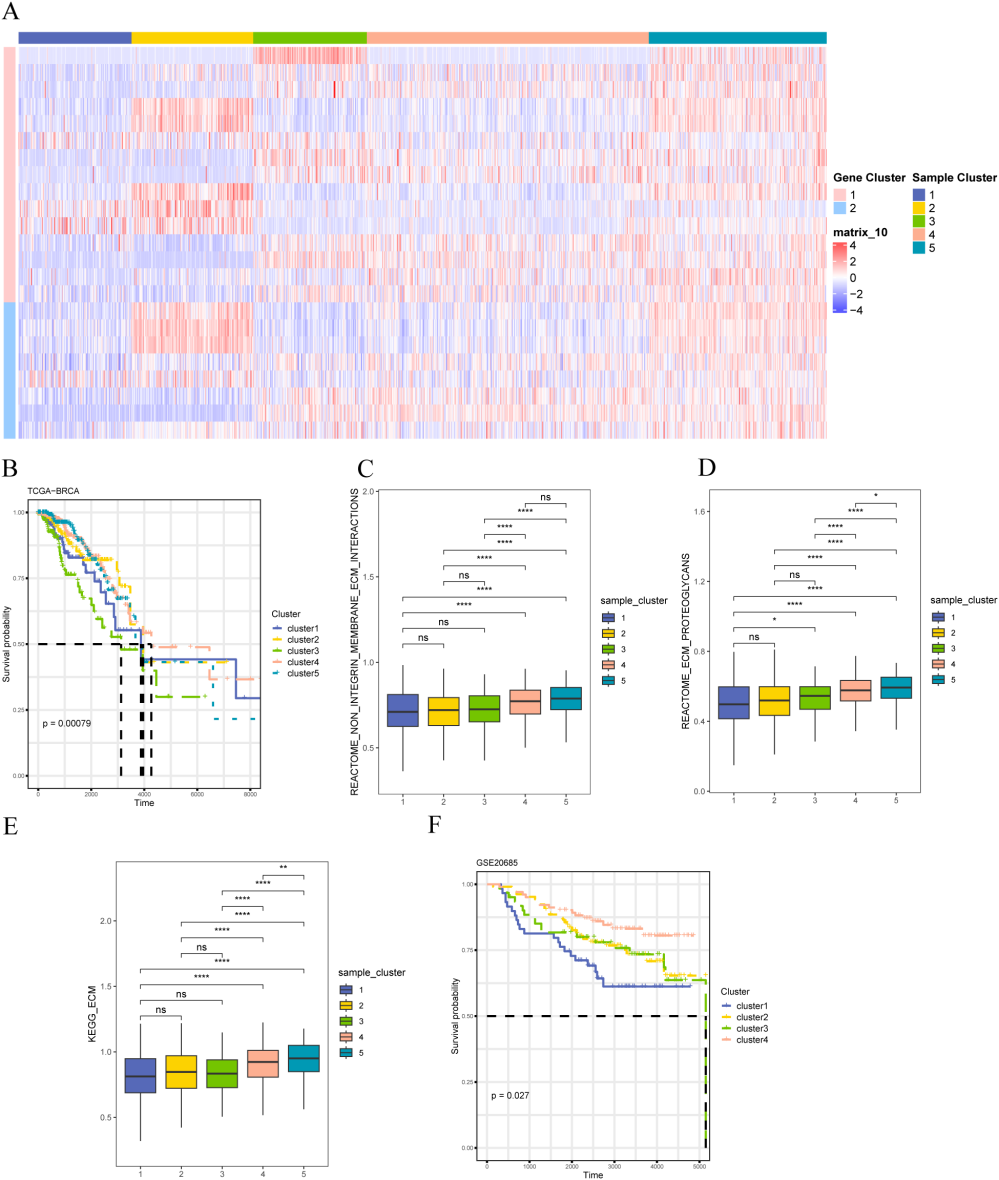
Identification of stromal molecular subtypes in breast cancer **(A) **Expression heat map of differentially expressed stromal body-related genes; **(B)** K-M survival curves of patients with different stromal molecular subtypes in the TCGA-BRCA dataset; **(C)** REACTOME ECM Interactions of patients with different stromal molecular subtypes Enrichment degree of pathway; **(D)** enrichment degree of REACTOME ECM Proteoglycans pathway in patients with different matrix molecular subtypes; **(E)** enrichment degree of KEGG_ECM pathway in patients with different matrix molecular subtypes; **(F)** GSE20685 K–M survival curves for patients with different matrix molecular subtypes in the dataset. The symbol ns is equivalent to p≥0.05, no statistical significance; the symbol * is equivalent to p< 0.05; the symbol ** is equivalent to p< 0.01; **** is equivalent to p < 0.0001.

We used a consensus clustering algorithm based on a Monte Carlo reference (Monte Carlo reference-based consensus clustering, M3C) to identify the matrix-associated molecular subtypes based on the expression of matrix body-associated DEGs (MRDEGs). Five matrix-associated molecular subtypes were identified (**Figure 7A**). Survival analysis revealed that the five distinct stromal-associated molecular subtypes had significantly different survival rates, with patients in Cluster 3 having the worst prognosis (**Figure 7B**). To further explore the underlying molecular mechanism, Cluster 5 samples were significantly enriched in ECM interactions (**Figure 7C**), ECM proteoglycans (**Figure 7D**), and KEGG_ECM (**Figure 7E**) pathways, indicating that Cluster 5 samples had higher matrix body-related activity. To validate the feasibility of our clustering results, we performed the same clustering on the GSE20685 dataset and found that the samples clustered into four categories, with significant differences in survival. The lack of significance in cluster 4 may be due to the biological characteristics of the samples in this category, the small sample size, or the heterogeneity of clinical features. This indicates that the prognosis of patients in cluster 4 is relatively uniform, with no obvious survival differences. Additionally, the gene expression or related molecular pathways in this category may not have had a significant impact on patient prognosis, thus failing to achieve statistical significance in the survival analysis (**Figure 7F**).

**Figure 8:**
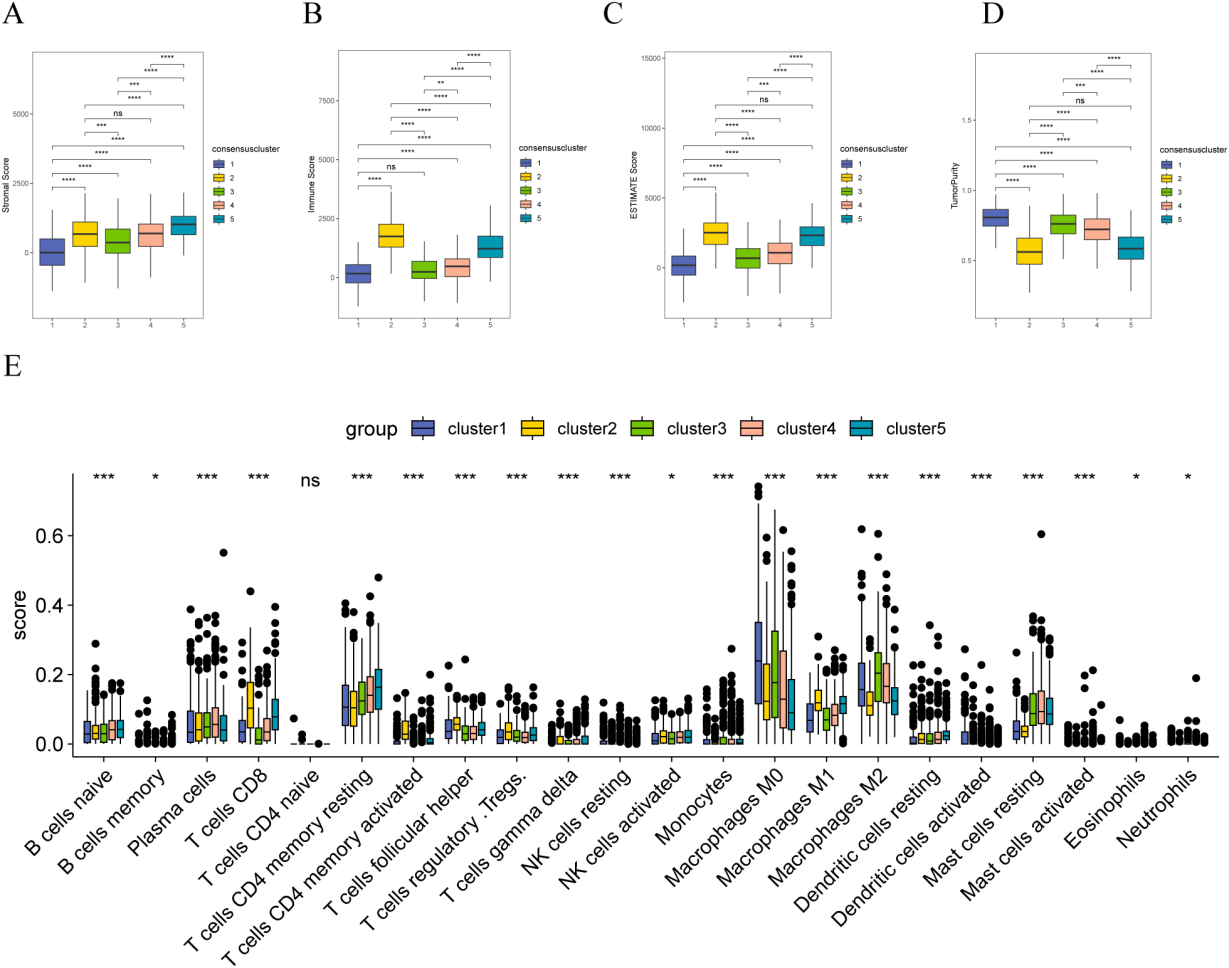
Immune correlation analysis of stromal molecular subtypes in breast cancer **(A)** Distribution of matrix scores in patients with different matrix molecular subtypes; **(B)** Distribution of immune scores in patients with different matrix molecular subtypes; **(C)** Distribution of ESTIMATE scores in patients with different matrix molecular subtypes; **(D)** Tumor purity of patients with different stromal molecular subtypes; **(E)** distribution of infiltration degree of 22 immune cell types in patients with different stromal molecular subtypes; the symbol ns is equal to p ≥ 0.05, no statistical significance; the symbol * is equal to p < 0.05; the symbol ** is equivalent to p < 0.01; the symbol *** is equivalent to p < 0.001; the symbol **** is equivalent to p < 0.0001.

Furthermore, we analyzed the differences in immunity between the different sample clusters. The ESTIMATE analysis indicated that Cluster 2 samples had the highest matrix (**Figure 8A**), immune (**Figure 8B**), and ESTIMATE (**Figure 8C**) scores and exhibited the lowest tumor purity (**Figure 8D**). Conversely, Cluster 1 showed the highest tumor purity. The CIBERSORT analysis revealed significant differences in the infiltration of various immune cells among patients with different molecular subtypes. Notably, CD8+ T cells and activated NK cells showed higher enrichment in Cluster 2 samples but lower enrichment in those of Cluster 1; however, T cells CD4 memory resting cells are more enriched in Cluster-5 samples and less enriched in Cluster-2 samples. Macrophages M0 cells are more enriched in Cluster-1 samples and less enriched in Cluster-5 samples. Macrophages M2 cells are more enriched in Cluster-3 samples and less enriched in Cluster-2 samples (**Figure 8E**).

**Figure 9:**
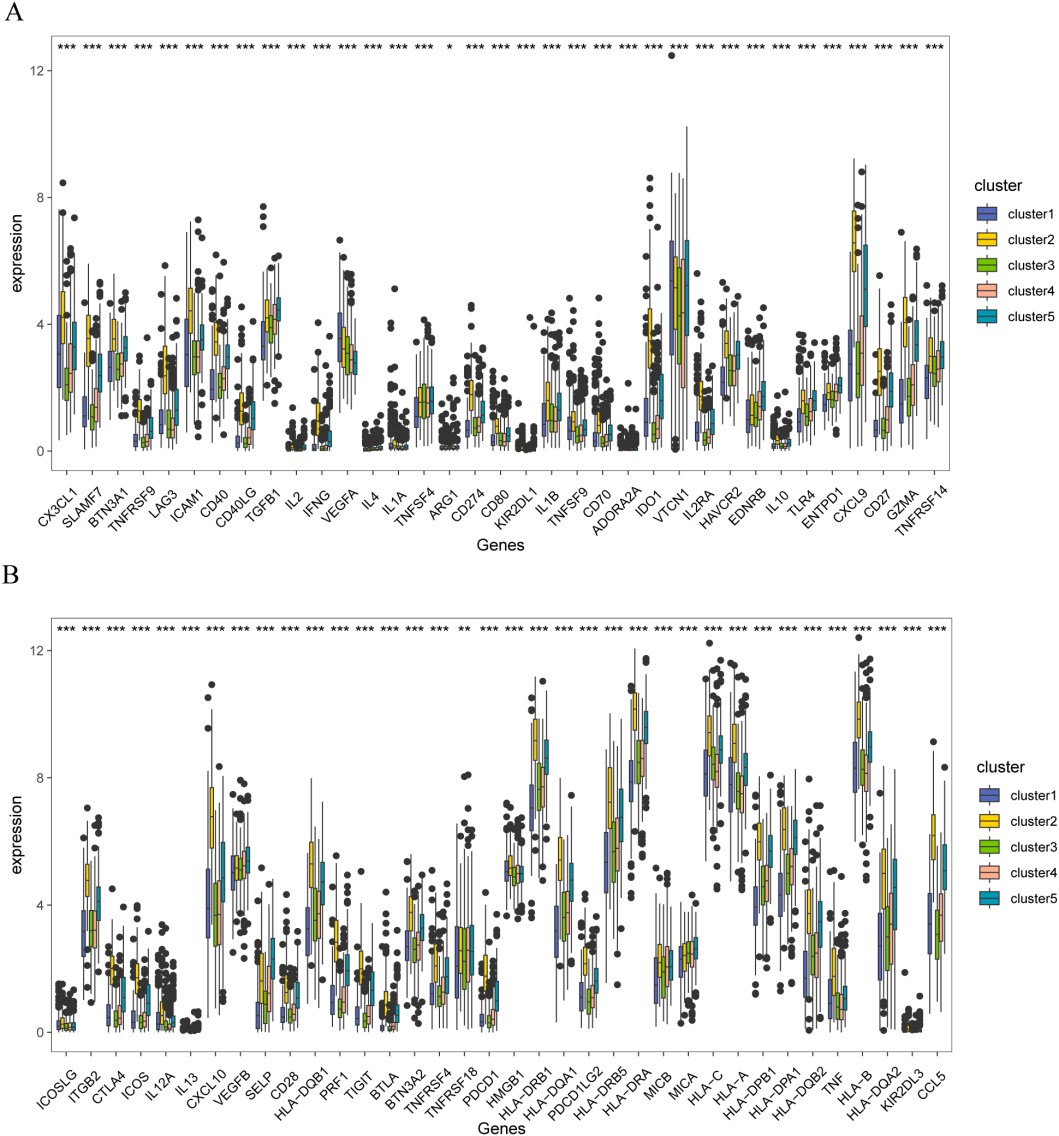
Immune checkpoint correlation analysis of stromal molecular subtypes in breast cancer **(A)** Distribution of immune checkpoint gene expression in patients with different matrix molecular subtypes, **(B)** Distribution of immune checkpoint gene expression in patients with different matrix molecular subtypes. The symbol ns is equivalent to p≥ 0.05, no statistical significance; the symbol * is equivalent to p< 0.05; the symbol ** is equivalent to p< 0.01; the symbol *** is equivalent to p< 0.001.

Furthermore, significant differences were observed in the expression of immune checkpoint genes among patients with the five molecular subtypes (**Figures 9A, B**). These results indicate that stromal body genes may affect the prognosis of patients with breast cancer by regulating their immune response and infiltration.

### Gene mutation analysis of stromal molecular subtype populations in different breast cancers

**Figure 10:**
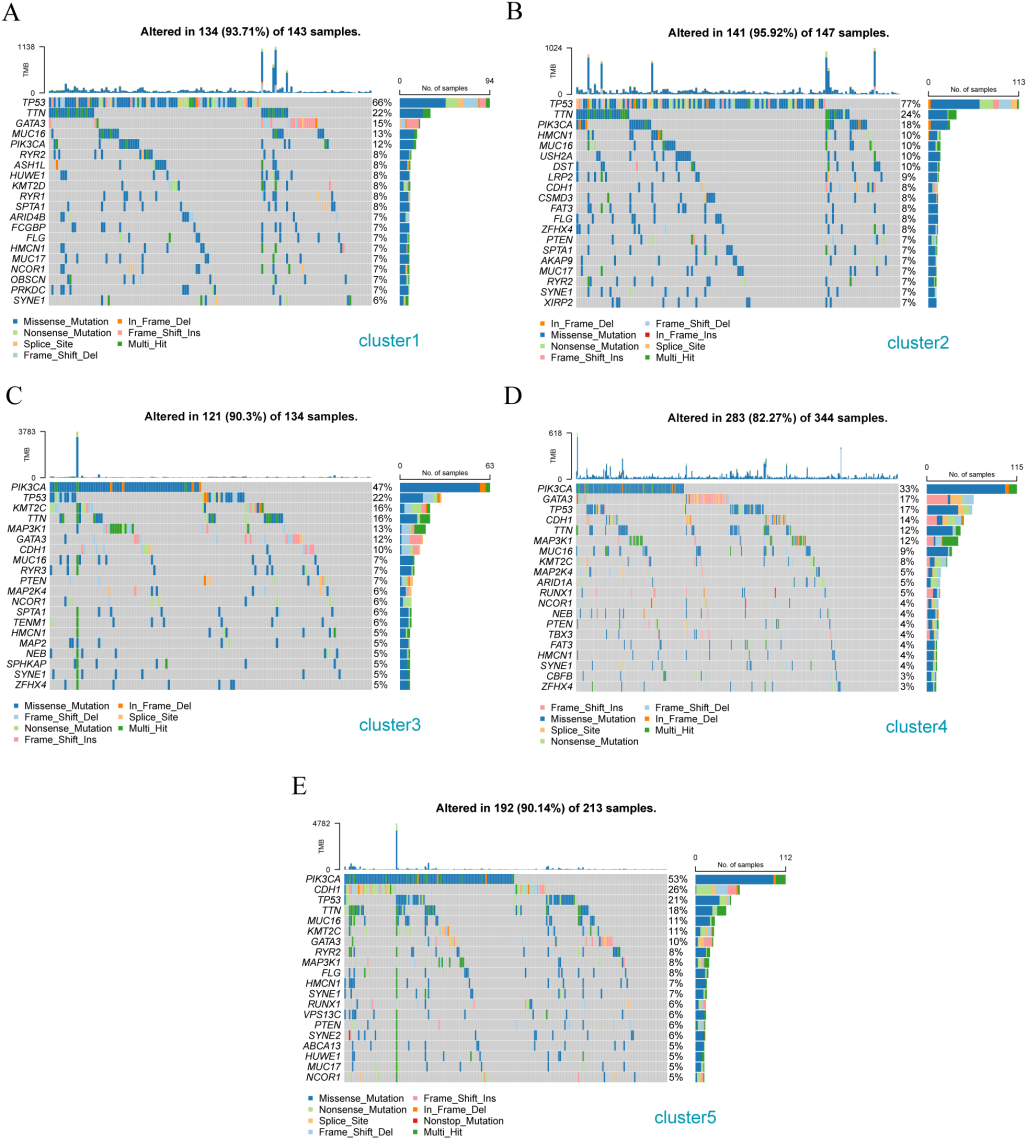
Gene mutation analysis of different breast cancer subtypes. **(A)** Gene mutation waterfall diagram of Cluster -1 sample cluster; **(B)** Gene mutation waterfall diagram of Cluster -2 sample cluster; **(C)** Gene mutation waterfall diagram of Cluster -3 sample cluster; **(D)** Gene mutation waterfall diagram of Cluster - 4 sample clusters; **(E)** Cascade diagram of gene mutations in Cluster - 5 sample clusters.

The mutation characteristics of the above stromal-associated breast cancer subgroups were analyzed using the R package maftools. The Cluster 1 subtype primarily had mutations in *TP53*, *TTN*, and *GATA 3* (**Figure 10A**); the Cluster 2 subtype primarily had *TP53, TTN*, and *PIK3CA* mutations (**Figure 10B**); the Cluster 3 subtype primarily had *PIK3CA, TP53*, and *KMT2C* gene mutations (**Figure 10C**); the Cluster 4 subtype primarily exhibited *PIK3CA, GATA3*, and *TP53* gene mutations (**Figure 10D**); the Cluster 5 subtype primarily had *PIK3CA, CDH1*, and *TP53* gene mutations (**Figure 10E**).

**Figure 11:**
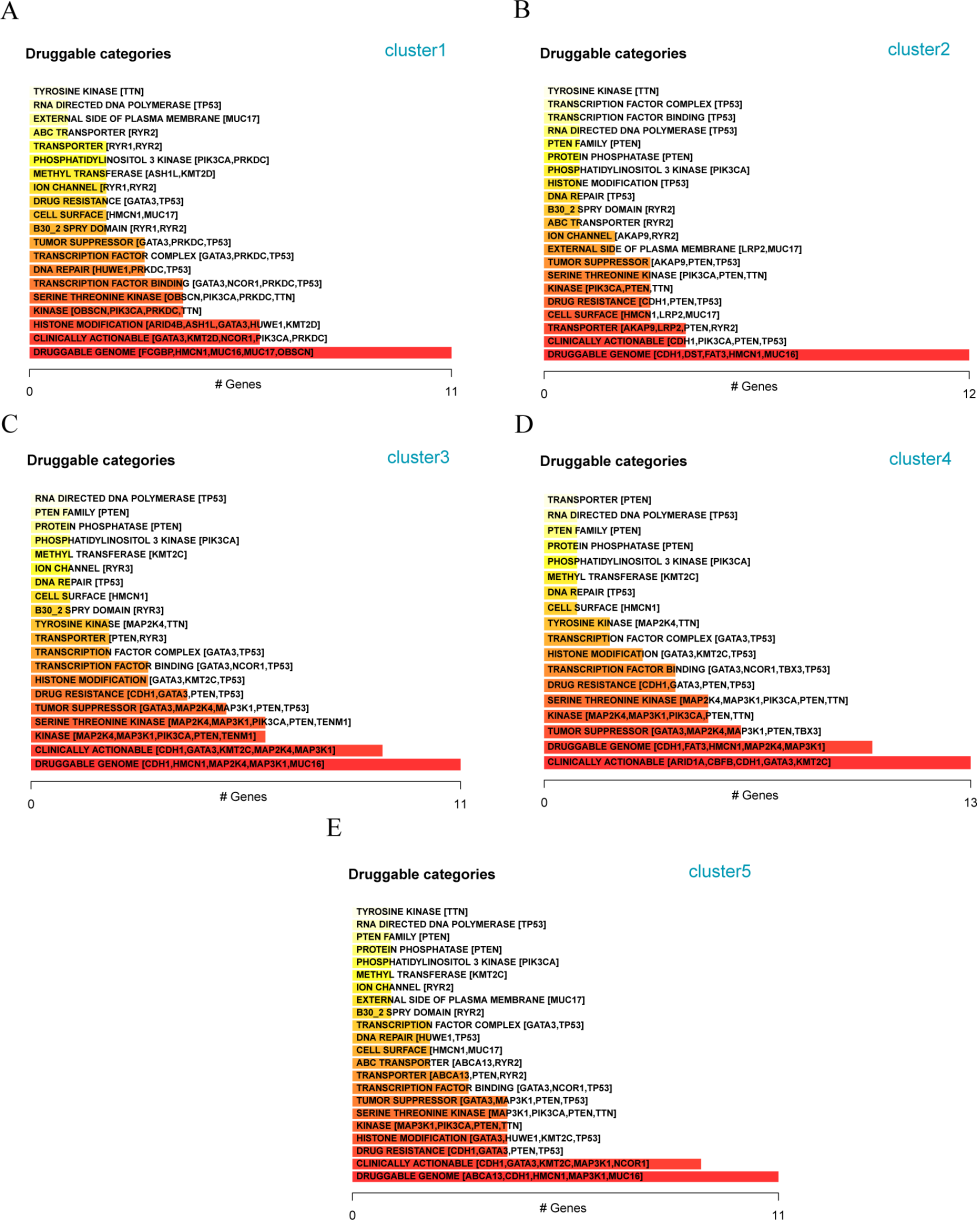
Analysis of available genes of matrix molecular subtypes in different breast cancer populations **(A)** Classification of potentially druggable genes in Cluster-1; **(B)** Classification of potentially druggable genes in Cluster-2; **(C)** Classification of potentially druggable genes in Cluster-3; **(D)** Classification of potentially druggable genes in Cluster-4; **(E)** Classification of potentially druggable genes in Cluster-5.

Furthermore, we analyzed the mutations of the three patient subtypes to explore the gene druggability and the interaction between drugs and genes (from Drug Gene Interaction database, DGIdb database) and found that the genes to predict that the drug might act on Cluster 1, 2, 3, 4, and 5 subgroups are DRUGGABLE GENOME (*FCGBP, HMCN1, MUC16, MUC17*, and *OBSCN*) (**Figure 11A**), DRUGGABLE GENOME (*CDH1, DST, FAT3, HMCN1*, and *MUC16*) (**Figure 11B**), DRUGGABLE GENOME (*CDH1, HMCN1, MAP2K4, MAP3K1, and MUC16*) (**Figure 11C**), CLINICALLY ACTIONABLE (*ARID1A, CBFB, CDH1, GATA3*, and *KMT2C*) (**Figure 11D**), and DRUGGABLE GENOME (*ABCA13, CDH1, HMCN1, MAP3K1*, and *MUC16*) (**Figure 11E**), respectively, indicating that these mutated genes can be used for subsequent studies on the development of drug targets.

**Figure 12:**
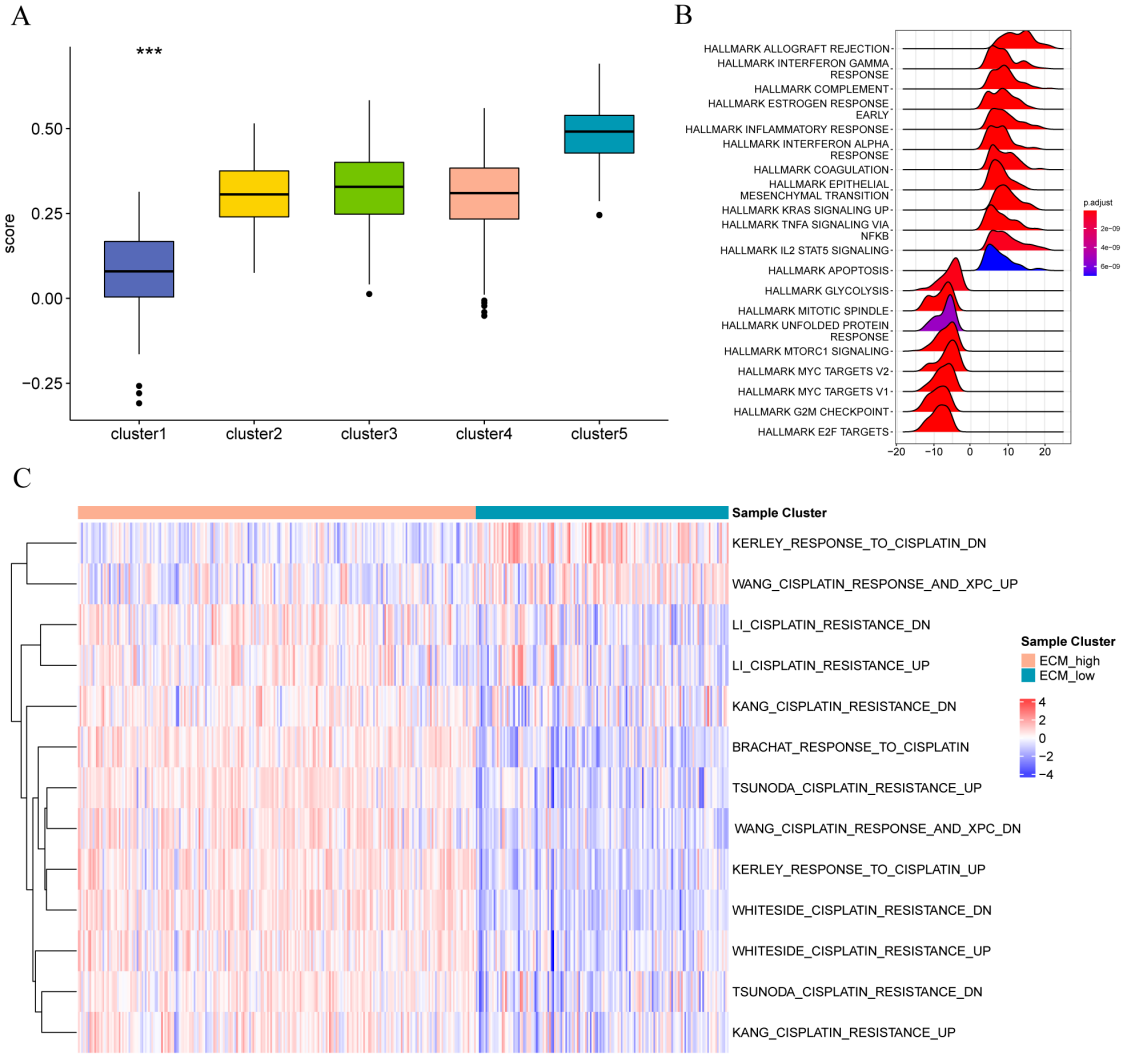
Functional enrichment analysis of stromal molecular subtype populations in different breast cancers **(A)** Group comparison diagram of matrix scores of different subtypes of samples; **(B)** enrichment of Hallmark gene set among different sample groups; **(C)** enrichment of C2 oncogenic pathway gene set among different sample groups.

Subsequently, we calculated the scores of marker genes related to the matrix (*CCL5, CLEC3A, CST1, CST7, CTSW, CXCL9, EMID1, HPX, IGFALS, LAMB3, PLAT, S100B, SCUBE2, SEMA3B*, and *SERPINA1*) using the ssGSEA algorithm. Comparing the **Figure 12A** score of different typing scores of samples with the sample typing information revealed that the Cluster 1 score was the lowest; we defined it as the ECM-low group. Cluster 5 score was the highest, and we defined it as the ECM-high group. The hallmark (**Figure 12B**), and C2 (**Figure 12C**) enrichment pathway analyses for different patient groups, the colors in the heatmap indicate the relative expression levels: red for high expression, blue for low expression. The clustering on left shows the hierarchical relationship of samples based on their gene expression profiles. It revealed that the ECM-high group samples were primarily enriched in APOPTOSIS, HALLMARK IL2 STAT5 SIGNALING, HALLMARK TNFA SIGNALING VIA NFKB, HALLMARK KRAS SIGNALING UP, HALLMARK EPITHELIAL MESENCHYMAL TRANSITIO, HALLMARK COAGULATION, HALLMARK INTERFERON ALPHA RESPONSE, HALLMARK INFLAMMATORY RESPONSE, HALLMARK ESTROGEN RESPONSE EARLY, HALLMARK COMPLEMENT, HALLMARK INTERFERON GAMMA RESPONSE, and HALLMARK ALLOGRAFT REJECTION, and the ECM-low group samples are primarily enriched in LI_CISPLATIN_RESISTANCE_DN, LI_CISPLATIN_RESISTANCE_UP, KANG_CISPLATIN_RESISTANCE_DN, and BRACHAT_RESPONSE_TO_CISPLATIN. Apoptosis, IL2 stat5 signaling, Tnfa signaling via NFKB, Kras signaling up, Epithelial-mesenchymal transition, Coagulation, Interferon alpha response, Inflammatory response, Estrogen response early, Complement, Interferon-gamma response, Allograft rejection, and the ECM-low group samples are mainly enriched in Cisplatin resistance dn, Cisplatin resistance up, Kang cisplatin resistance dn, and Brachat response to cisplatin.

**Figure 13:**
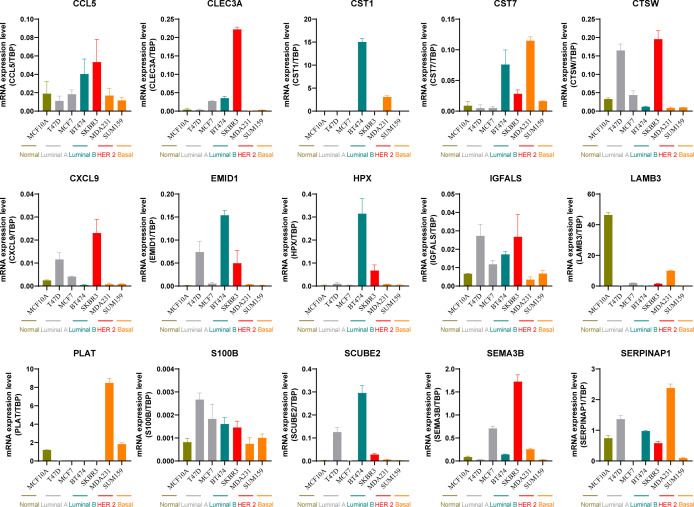
Expression of matrix related marker genes in different breast cancer cells.

Similarly, we assessed the expression of matrix-related marker genes (*CCL5, CLEC3A, CST1, CST7, CTSW, CXCL9, EMID1, HPX, IGFALS, LAMB3, PLAT, S100B, SCUBE2, SEMA3B*, and *SERPINA1*) in various breast cancer subtypes (**Figure 13**). The results showed that different markers, such as *CTSW* and *S100B*, were specifically overexpressed in Luminal A breast cancer cells. *CST1, EMI D1*, and *HPX SCUBE2* were specifically overexpressed in Luminal B breast cancer cells. *CCL5, CLEC3A, CTSW, CXCL9, IGFALS*, and *SEMA3B* were specifically overexpressed in HER2-positive breast cancer cells. *CST7, PLAT*, and *SERPINA1* were specifically overexpressed in Basal-like breast cancer cells.

### Cell composition analysis of the ECM-high and -low groups

**Figure 14:**
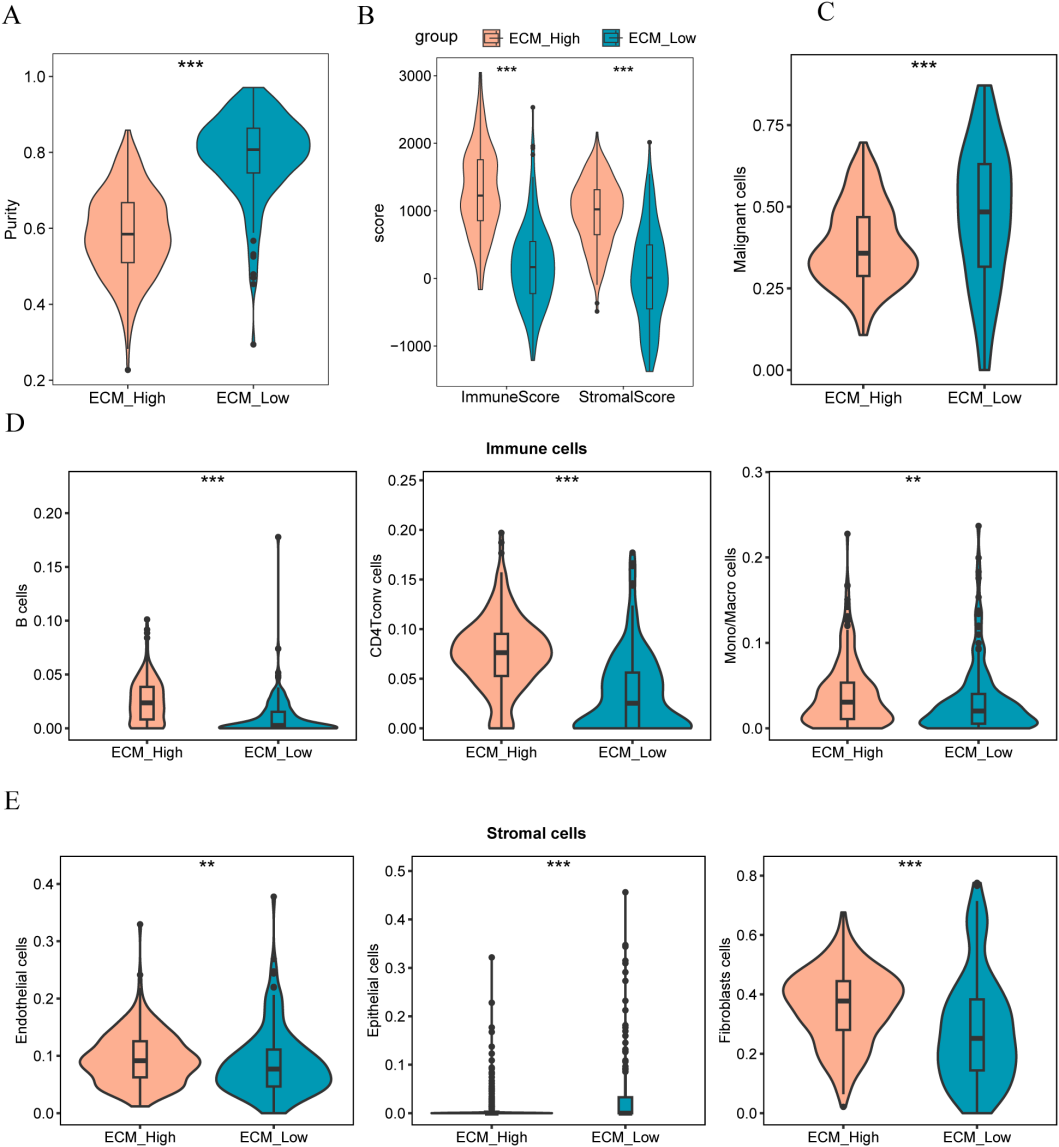
**(A, B)** Comparison charts of tumor purity, stroma score, and immune score in high and low ECM group samples; **(C)** Comparison chart of malignant tumor scores in high and low ECM group samples; **(D)** Comparison chart of scores in different immune cells for high and low ECM group samples; **(E)** Comparison chart of scores in different stromal cells for high and low ECM group samples. The symbol ** indicates p < 0.01, which has high statistical significance; the symbol *** indicates p < 0.001, which has extremely high statistical significance.

We used ESTIMATE to assess tumor purity between different groups (ECM-high vs. ECM-low), revealing a notable difference in the tumor purity between them (p<0.05, **Figure 14A**). Furthermore, the ECM-high group exhibited lower tumor purity owing to its higher matrix and immune scores (p<0.05; **Figure 14B**). Moreover, we developed a reference matrix for CIBERSORTx using the cell types identified in the single-cell dataset (GSE161529). Deconvolution methods were used to calculate the scores of TCGA-BRCA samples for different cell types.

The findings revealed that samples from the ECM-low group exhibited higher malignant tumor scores (**Figure 14C**). Conversely, samples from the ECM-high group had a significant enrichment of immune cells, particularly B cells, macrophages, monocytes, and CD4 T cells (**Figure 14D**). Furthermore, the ECM-high group samples demonstrated significant enrichment of stromal cells, specifically endothelial cells and fibroblasts, whereas the ECM-low group samples were notably enriched in epithelial cells (**Figure 14E**).

### Ligand-receptor interaction analysis

**Figure 15:**
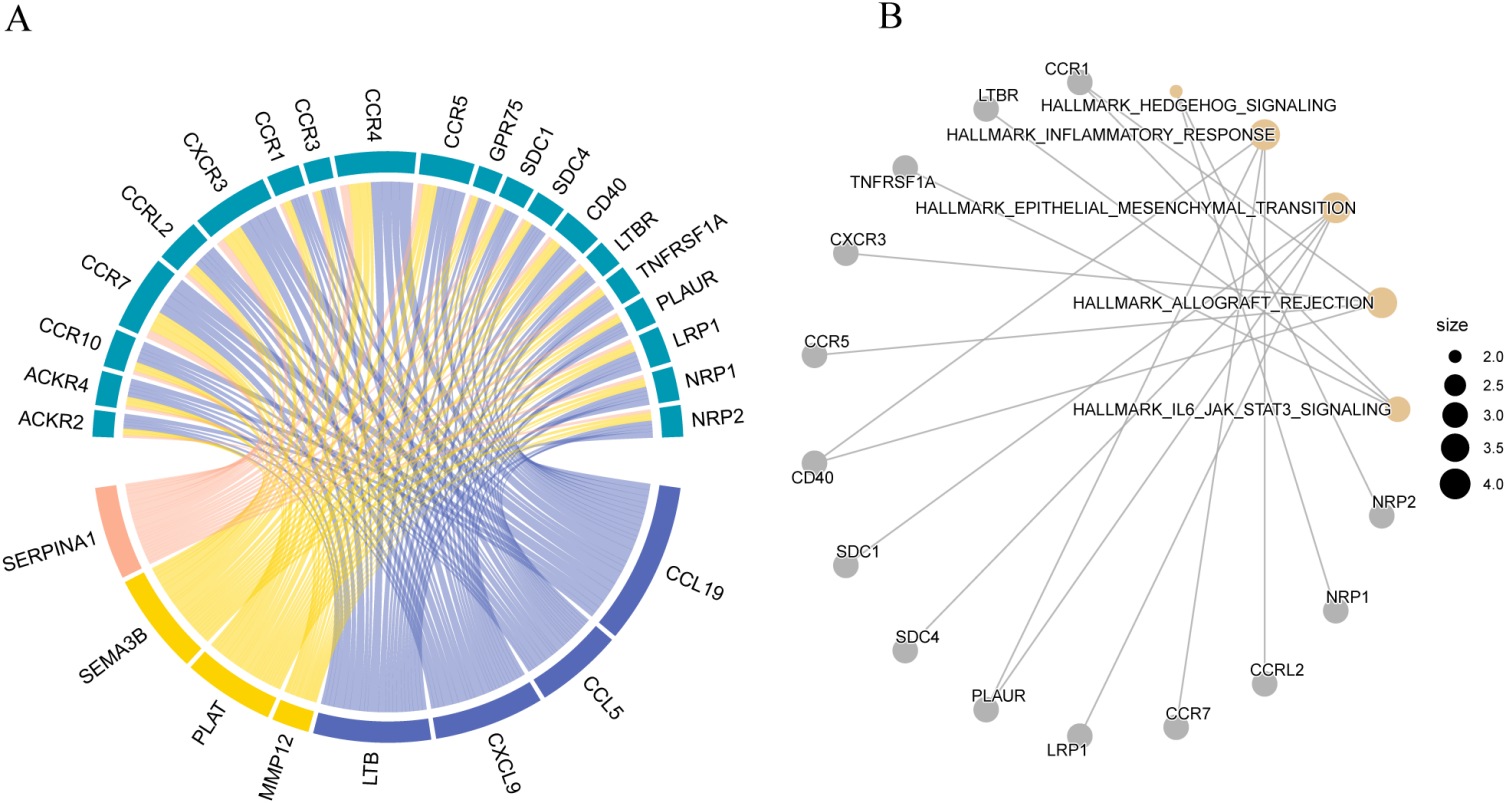
Ligand-receptor interaction analysis **(A)** Receptor-ligand interaction diagram; **(B)** Enrichment analysis of Hallmark pathways corresponding to matrix ligands and their receptors.

ECM components interact directly with cell surface receptors, regulating the activity of numerous signaling pathways, including those related to epithelial-mesenchymal transition (EMT) and ECM production. We conducted a ligand-receptor interaction analysis to elucidate the potential direct effects of these maturation forms on cell signaling. The results showed that the matrix body genes *CCL19, CCL5, CXCL9, LTB, MMP12, PLAT, SEMA3B*, and *SERPINA1* interacted with many receptors (**Figure 15A**). Interacting receptors are crucial in cancer development, participating in the IL-6/JAK/STAT 3 signaling pathway (**Figure 15B**).

## Discussion

Breast cancer is the most common cancer worldwide and is critical to human life and health. Understanding the behavioral mechanisms of breast cancer cells could provide better coping strategies for treatment; however, the behavior of tumor cells is complex. Owing to the advancement in the literature, researchers have suggested that cell behavior should be studied based on the internal mechanisms of cells and the situation of the cell matrix. Biological tissues comprise cells and the ECM. The ECM is a three-dimensional scaffold (42) that supports the activities and microenvironment of the whole cell and promotes the biological signal transmission of tissue cells. Researchers have considered this as an essential aspect of regulating the microenvironment of cell behavior and phenotypes. Research has found a close relationship between matrix genes and breast cancer (43). However, the relationship between breast cancer matrix genes and the prognosis of breast cancer has no targets and mechanisms, and the relationship between matrix genes and immune invasion is also unclear. Therefore, we aimed to analyze the relevant characteristics of matrix genes in breast cancer through the multigroup data of a breast cancer multi-database, identify 127 differential genes of breast cancer matrix genes using the elastic net penalty logic regression method, and construct the risk characteristics of matrix genes in breast cancer. This model could be used to reasonably predict the prognosis of breast cancer. Subsequently, a consensus clustering algorithm was used to identify matrix molecular subtypes in breast cancer, and five matrix-related molecular subtypes were identified. The biological characteristics of the matrix molecular subtypes in different breast cancers were determined through gene mutation, immune correlation, pathway, and ligand-receptor analyses. Similarly, we used ssGSEA to compute the expression levels of 15 marker genes associated with the matrix. Subsequently, we assessed the expression of these marker genes in different breast cancer cell lines using qPCR. We found that the gene expression and immune invasion of various breast cancer matrix molecular subtypes differed significantly. Our analysis revealed the biological characteristics of matrix genes in breast cancer subtypes and guided future studies on improving the diagnosis and treatment of patients with breast cancer with poor prognosis.

Through differential gene analysis and a penalty logic regression algorithm of the elastic net, 15 stromal cell-related marker genes were identified. Among them, *CLEC3A* was highly expressed in patients with estrogen-positive breast cancer (42), the expression of CLEC3A is significantly associated with the overall survival of patients, and other studies (44) found that *CLEC3A* was associated with immune invasion of lung squamous cell carcinoma. *CCL5* is a chemokine involved in the activation of CD8+ T cells, and its expression influences the immune infiltration of breast cancer cells (45), CCL5 is closely related to disease-free survival. *SEMA3B* is associated with glioblastoma multiforme (46), uveal melanoma (47), breast cancer (48), gastric cancer (49), and other tumors. *EMID1* is more than lung cancer and lung injury (50); however, no study has found a relationship between *CXCL9* and breast cancer. Some studies (51) have identified *CXCL9* as a T cell chemokine related to the prognosis of head and neck cancer (51), prostate cancer (52), melanoma (53), ovarian cancer (54), gastric cancer (55) and other tumors, and studies primarily focus on the immune infiltration of CD8 T cells. *S100B* is primarily associated with neurological tumors in children (56). Studies have shown that *S100B* is a good predictor of disease-free survival of breast cancer (57). Several studies have demonstrated a close association between *SERPINA1* and digestive tract tumors, indicating a strong correlation with tumor-infiltrating lymphocytes. However, to date, no study has established a relationship between *SERPINA1* and breast cancer. We concurrently assessed the expression of stromal-related marker genes across different breast cancer subtypes. These findings reveal the differential expression of various stromal marker genes across different breast cancer subtypes. For example, *CST1* is highly expressed in Luminal B breast cancer cells, *SEMA3B* is highly expressed in HER2-positive breast cancer cells, while SEMA3B stands out in the analysis of progression-free survival, and *PLAT* is highly expressed in basal-like breast cancer cells. This suggests that specific clinical decisions should be made according to the breast cancer type. The significant expression of these genes in different subtypes suggests their potential in subtype-specific therapies.

This model serves as a robust tool to predict the survival and prognosis of patients with breast cancer. Our model significantly improves the accurate prediction of prognosis in breast cancer patients by integrating the expression characteristics of matrix-related genes. Especially in assessing the impact of tumor infiltration and the immune microenvironment, this model demonstrates strong predictive capability.

Furthermore, it is crucial in tumor immunity, offering a novel avenue for assessing the immune status of patients and guiding immunotherapy selection.

Firth’s correction was used to calculate the risk ratio (odds ratios) for each marker gene to determine the risk of generating matrix-based genes in the sample. Based on the matrix risk scores of patients in the dataset, individuals were stratified into high- and low-risk groups. The Kaplan–Meier test was used to compare the overall survival differences between the different sample groups, revealing a statistically significant difference between the two groups. The low-risk group exhibited a significantly longer survival period than the high-risk group. The matrix risk score was notably higher in deceased and older patients with breast cancer and lower in patients with T1 breast cancer. To validate the efficiency of our model, it was applied to the GSE20685 and UCSC Caldas2007 datasets. Similarly, this model could be used to significantly separate patients with breast cancer with different prognoses in the dataset. Therefore, the devised stromal gene model in this study can serve as a robust model for predicting the survival and prognosis of patients with breast cancer.

More matrix genes have been identified than tumor immune cells in previous studies. We assessed tumor purity in different groups (ECM-high and ECM-low). The results demonstrated a notable disparity in tumor purity between the ECM-high and ECM-low groups, with lower tumor purity observed in the ECM-high group, attributed to its higher matrix and immune scores. Moreover, we constructed a reference matrix of CIBERSORTx and calculated the scores of TCGA-BRCA samples in different cell types using the deconvolution method. The results indicated higher malignant tumor scores in samples from the ECM-low group. The samples in the ECM-high group exhibited a significant enrichment of immune cells, particularly B cells, macrophages/monocytes, and CD4 T cells. In contrast, samples from the ECM-high group showed significant enrichment of endothelial cells and fibroblasts in the matrix cell population. ECM-low was significantly enriched in epithelial cells. In tumor immunotherapy, researchers divide tumor immune cell infiltration into “hot tumor” and “cold tumor.” “Hot tumor” has an excellent response to immunotherapy (58, 59). Therefore, converting a “cold tumor” into a “hot tumor” has been focused on by researchers. Some breast cancer patients often face systemic toxicity and low response rates when undergoing immunotherapy, primarily due to the immunosuppressive tumor microenvironment. Therefore, reversing the immunosuppressive tumor microenvironment is considered crucial for enhancing the efficacy of immunotherapy. As researchers explore ways to reverse immunosuppression, some have utilized bioorthogonal click chemistry and PD-L1 targeted imaging (60). It has been found that the expression of necroptosis-related genes is closely associated with immune cell infiltration and the activation of immune checkpoints, suggesting that guiding personalized treatment strategies based on necroptosis characteristics could improve the prognosis and treatment outcomes for breast cancer patients (61). Additionally, researchers have discovered that oxidative stress-related genes play a significant role in regulating the behavior of tumor cells and immune cells, thereby affecting tumor progression and prognosis (62). Our results indicate that the expression of matrix genes influences the infiltration of tumor immune cells. Improving the state of tumor immune infiltration by interfering with the expression of matrix genes could also affect the prognosis of patients with tumors.

To explore which cell types express the marker genes that we identified to construct our matrix extraction risk characteristics, based on the cell annotation information of a single-cell dataset (GSE161529), we calculated the enrichment degree of matrix risk genes with positive (positive score) and negative (negative score) ratios in specific cell types and found that in negative scores, CD4 Tconv and endothelial, epithelial, fibroblast, malignant, mono/macro, and pericyte, and plasma cells differed significantly between tumor and normal cells. In the positive score, CD4T conv and endothelial, epithelial, fibroblast, malignant, mono/macro, NK, pericyte, and plasma cells significantly differed between tumor and normal cells.

Further analysis revealed significant differences in the expression of immune checkpoint genes among patients with five molecular subtypes. These results indicate that the matrix gene might affect the prognosis of patients with breast cancer by regulating the immune response and infiltration.

Analysis of gene mutations in different subtypes of stromal molecules in breast cancer revealed that *TP53* was commonly mutated across all five subtypes, and the commonly mutated gene was *PIK3CA*. *TP53* is also known as p53. Among the human genes, *TP53* is a critical tumor suppressor that exhibits low expression in normal cells and high expression in malignant tumors. The p53 protein encoded by *TP53* is a vital regulator of cell growth, proliferation, and repair in response to cellular damage. During the process of DNA damage, p53 halts the cell cycle at the G1/S phase boundary, facilitates DNA repair, and induces apoptosis if repair is not feasible (63). *PIK3CA* mutations occur in approximately 8% of cancers, including 40% of HR-positive breast cancers (64). It is a pan-cancer mutagen; therefore, studying *PIK3CA* is more conducive to the development of clinical drugs. In analyzing pharmaceutically available genes in populations with different matrix molecular subtypes of breast cancer, four groups of subtype gene populations contained the gene *HMCN1*, which encodes immunoglobulin. However, its role in humans remains unclear, but *HMCN* in Caenorhabditis elegans has multiple functions in transient cell contact required for cell migration and basement membrane invasion, and there is stable contact between the semi-chromosome-mediated cell junction and the elastic fibrous structure (65). Mutations in this gene have also been found in gastric and colorectal cancers (66). *HMCN1* was mutated in this study population of breast cancer; therefore, this gene can be used as a target for drug development in the future. Another mutated gene is *MUC16*, mucin 16, also known as a cancer antibody 125 (CA125). *MUC16* is implicated in various tumor signaling pathways, including those in ovarian (67), breast (68), cervical (69), pancreatic (70), and colorectal (71) cancers. Elevated *MUC16* expression is correlated with cancer progression, metastasis, and poor prognosis in patients.

The genetic constituents of the matrix directly engage cell surface receptors, modulating the activity of numerous signaling pathways. Through ligand-receptor analysis, we found that the matrix marker genes primarily acted on the inflammatory response, EMT, and IL-/JAK/STAT3 signaling pathway. Previous studies have found that inflammation stimulates tumor cells, promotes their growth, and alters the tumor microenvironment (72, 73). EMT is a biological process involving epithelial cell transition to acquire a mesenchymal phenotype through a defined program. During EMT, epithelial cells relinquish their characteristic epithelial traits, such as cell polarity and adhesion to the basement membrane, and acquire mesenchymal characteristics, such as enhanced migratory and invasive capabilities, resistance to apoptosis, and extracellular matrix degradation. EMT is a critical biological process that enables the migration and invasion of malignant tumor cells derived from epithelial origins (74, 75). This study shows that the ligands of matrix genes are mostly concentrated in the inflammatory signaling pathway and EMT, guiding the follow-up treatment and the development of corresponding drugs.

However, our study has some limitations. First, to fully clarify the influence of matrix genes on the prognosis of patients with breast cancer, microarray samples from different types of breast cancers are needed. Second, although we conducted several analyses in this study, such as using the ESTIMATE algorithm to assess the immune characteristics of the tumor microenvironment and employing CIBERSORT to analyze the composition of immune cell infiltration, to explore the role of smatrix genes in breast cancer and their relationship with the immune microenvironment, there are still some limitations. Although our results support the association between matrix genes and breast cancer prognosis through various public databases, the characteristics of many matrix genes in breast cancer are not clear, and the biological functions of these stromal marker genes in breast cancer require further verification, as there are no corresponding clinical correlation studies. These directions should be the focus of future studies.

## Conclusions

This comprehensive examination of cell-matrix genes in patients with breast cancer revealed the key genes influencing breast cancer prognosis. By integrating multiple omics datasets, we established a predictive model capable of forecasting the survival and prognosis of patients with breast cancer. In addition, the model is significant in tumor immunity, providing new directions for patient immune status assessment and immunotherapy selection. Receptor analysis showed that matrix genes were primarily involved in the inflammatory pathway. This study offers a novel foundation for clinical research and drug development in breast cancer to enhance the prognosis of patients with breast cancer.

## Data Availability

The original contributions presented in the study are included in the article/**Supplementary Material**. Further inquiries can be directed to the corresponding authors.

## Footnotes

1
https://portal.gdc.cancer.gov/

2
http://www.gseamsigdb.org/gsea/msigdb/search.jsp)

3 (http://tisch.comp-genomics.org/)

4 (http://xena.ucsc.edu/)

5
https://cibersortx.stanford.edu/

6
https://www.r-project.org , version 4.0.2
