# Supplementary material for: Single-cell analysis of matrisome-related genes in breast invasive carcinoma: new avenues for molecular subtyping and risk estimation
Source: Front Immunol. 2024 Oct 18;15:1466762. doi: 10.3389/fimmu.2024.1466762 (PMC11530991; doi:10.3389/fimmu.2024.1466762)
Supplement: Supplementary file 2 [file Table1.docx]

| Table 1 Datasets accessed in this study | | | | |
| --- | --- | --- | --- | --- |
| **Cohort** | **Data type** | **Source** | **Reference** | |
| TCGA-BRCA | RNAseq | TCGAbiolinks | | [1], [2] |
| Breast cancer | RNAseq | Gene Expression Omnibus GSE20685 | | [3] |
| Breast cancer | RNAseq | UCSC xene | | [4] |
| Cell types from scRNAseq | scRNAseq | h5 files and Signature Matrix | | [5] |
| **data type** |  |  | |  |
| Extracellular matrix gene set | Gene | Manuscript | | [6] |
| Cell types | scRNAseq | TISCH database | | [7] |

1. Silva, T.C., et al., *TCGA Workflow: Analyze cancer genomics and epigenomics data using Bioconductor packages.* F1000Res, 2016. **5**: p. 1542.

2. Zhang, Z., et al., *A survey and evaluation of Web-based tools/databases for variant analysis of TCGA data.* Brief Bioinform, 2019. **20**(4): p. 1524-1541.

3. Kao, K.J., et al., *Correlation of microarray-based breast cancer molecular subtypes and clinical outcomes: implications for treatment optimization.* BMC Cancer, 2011. **11**: p. 143.

4. Naderi, A., et al., *A gene-expression signature to predict survival in breast cancer across independent data sets.* Oncogene, 2007. **26**(10): p. 1507-16.

5. Pal, B., et al., *A single-cell RNA expression atlas of normal, preneoplastic and tumorigenic states in the human breast.* Embo j, 2021. **40**(11): p. e107333.

6. Naba, A., et al., *The matrisome: in silico definition and in vivo characterization by proteomics of normal and tumor extracellular matrices.* Mol Cell Proteomics, 2012. **11**(4): p. M111 014647.

7. Sun, D., et al., *TISCH: a comprehensive web resource enabling interactive single-cell transcriptome visualization of tumor microenvironment.* Nucleic Acids Research, 2020. **49**(D1): p. D1420-D1430.
