# Supplementary material for: Single-cell analysis of matrisome-related genes in breast invasive carcinoma: new avenues for molecular subtyping and risk estimation
Source: Front Immunol. 2024 Oct 18;15:1466762. doi: 10.3389/fimmu.2024.1466762 (PMC11530991; doi:10.3389/fimmu.2024.1466762)
Supplement: Supplementary file 3 [file Table2.docx]

**Table S2.** The primers of genes used in the article.

| Gene | Primer sequence (5' to 3') |
| --- | --- |
| TBP-F | TGCACAGGAGCCAAGAGTGAA |
| TBP-R | CACATCACAGCTCCCCACCA |
| CCL5-F | CAGTCGTCCACAGGTCAAGG |
| CCL5-R | CTTCTCTGGGTTGGCACACA |
| CLEC3A-F | CGAGGCACTAAAGTTCACAAGA |
| CLEC3A-R | CGGAGTTCCTGGGGATAACCA |
| CST1-F | GGTGGCATCTATAACGCAGAC |
| CST1-R | GTTGCCTGGCTCTTAGTACCC |
| CST7-F | GTGTGAAGCCAGGATTTCCTAA |
| CST7-R | TGTCGTTCGTGCAGTTGTTGA |
| CTSW-F | AGATCCAGTTCAACCGGAGTT |
| CTSW-R | TGTGAGGTCACTGAATGGAGT |
| CXCL9-F | CCAGTAGTGAGAAAGGGTCGC |
| CXCL9-R | AGGGCTTGGGGCAAATTGTT |
| EMID1-F | CGCACCATCTCATGCCATG |
| EMID1-R | CGGGCGGTCACTATCTTGT |
| HPX-F | CGTGACTGAACGCTGCTCA |
| HPX-R | CTCCCGGTCCCATTTGTGAC |
| IGFALS-F | CATTGCCCAACAGCCTCTTG |
| IGFALS-R | GTGAAGGTCCGCAGTGAGTT |
| LAMB3-F | GCAGCCTCACAACTACTACAG |
| LAMB3-R | CCAGGTCTTACCGAAGTCTGA |
| PLAT-F | TGCTGTGAAATAGATACCAGGGC |
| PLAT-R | TGAGTCTCGATCTGGGTTTCTG |
| S100B-F | TGGCCCTCATCGACGTTTTC |
| S100B-R | ATGTTCAAAGAACTCGTGGCA |
| SCUBE2-F | CCCACCTCCTACAAGTGCTC |
| SCUBE2-R | TGCAACGATAATTGCCTGGAAT |
| SEMA3B-F | ACATTGGTACTGAGTGCATGAAC |
| SEMA3B-R | GCCATCCTCTATCCTTCCTGG |
| SERPINA1-F | ATGCTGCCCAGAAGACAGATA |
| SERPINA1-R | CTGAAGGCGAACTCAGCCA |
